# Increased TRPV4 expression in non-myelinating Schwann cells is associated with demyelination after sciatic nerve injury

**DOI:** 10.1038/s42003-020-01444-9

**Published:** 2020-11-27

**Authors:** Xiaona Feng, Yasunori Takayama, Nobuhiko Ohno, Hirosato Kanda, Yi Dai, Takaaki Sokabe, Makoto Tominaga

**Affiliations:** 1 Department of Physiological Sciences, SOKENDAI, Okazaki, Japan; 2 Division of Cell Signaling, National Institute for Physiological Sciences, Okazaki, Japan; 3 Thermal Biology Group, Exploratory Research Center on Life and Living Systems (ExCELLS), Okazaki, Japan; 4 Department of Physiology, Showa University School of Medicine, Tokyo, Japan; 5 Division of Ultrastructural Research, National Institute for Physiological Sciences, Okazaki, Japan; 6 Department of Anatomy, Division of Histology and Cell Biology, Jichi Medical University, School of Medicine, Shimotsuke, Japan; 7 Department of Pharmacy, School of Pharmacy, Hyogo University of Health Sciences, Kobe, Japan

**Keywords:** Schwann cell, Neurophysiology

## Abstract

Transient receptor potential vanilloid 4 (TRPV4) is a non-selective calcium-permeable cation channel that is widely expressed and activated in various neurons and glial cells in the nervous system. Schwann cells (SCs) are primary glia cells that wrap around axons to form the myelin sheath in the peripheral nervous system. However, whether TRPV4 is expressed and functions in SCs is unclear. Here, we demonstrate functional expression of TRPV4 in mouse SCs and investigated its physiological significance. Deletion of TRPV4 did not affect normal myelin development for SCs in sciatic nerves in mice. However, after sciatic nerve cut injury, TRPV4 expression levels were remarkably increased in SCs following nerve demyelination. Ablation of TRPV4 expression impaired the demyelinating process after nerve injury, resulting in delayed remyelination and functional recovery of sciatic nerves. These results suggest that local activation of TRPV4 could be an attractive pharmacological target for therapeutic intervention after peripheral nerve injury.

## Abstract (web summary)

Feng et al. report that TRPV4 plays an important role in Schwann cells (SCs) during nerve demyelination and remyelination in mice. Using sciatic nerve cut injury mouse models, they find that TRPV4 expression is remarkably increased in demyelinating SCs during sciatic nerve injury; and ablation of TRPV4 expression impairs the demyelinating process after nerve injury, resulting in their delayed remyelination and functional recovery.

## Introduction

In the peripheral nervous system (PNS), myelin sheaths are formed by Schwann cells (SCs), which provide support and insulation to axons to facilitate rapid saltatory impulse conduction. Axonal sorting and myelination in PNS occur over an extended period during the first three weeks of postnatal life in rodents^1^. Immature SCs that contact small-diameter axons differentiate into non-myelinating SCs, which, in turn, contact large axons to form myelinating SCs. In contrast to the remarkable differentiation process that occurs during development, SCs remain highly plastic in order to respond adaptively to injury and trigger repair processes through cell type conversion^2^. Following a nerve cut or crush injury, the distal stump undergoes Wallerian degeneration. During this period, SCs play a key role. The distal nerve degenerates and are demyelinated while SCs acquire an immature-like phenotype termed “Bünger cells” or “repair SCs”, which are essential for nerve repair. Demyelinating SCs upregulate expression of several neurotrophic factors to promote neuronal survival and axon growth^3^, but also increase release of some cytokines that promote macrophage recruitment. Autophagic processes are activated in SCs^4–7^ that cooperate with these recruited macrophages to clear axon and myelin fragments that could inhibit axonal growth^4^. Repair SCs form tracks to guide regenerating axons back to their targets, whereupon SCs remyelinate.

A core set of transcription factors and signaling molecules are involved in SC differentiation during myelin development and remyelination following injury. Early growth response 2 (EGR2, Krox20) is a master positive regulator of myelination that promotes transcription of genes encoding myelin structural proteins as well as those involved in myelin lipid biosynthesis^8–11^. Many transcription factors are implicated in the control of myelination via regulation of Krox20. For example, Sox2 and c-Jun inhibit Krox20 expression that, in turn, impairs myelination^12,13^. Ca^2+^ signaling is another important mediator of myelination^14,15^ and regulates multiple intracellular pathways in SCs such as those involving the calcineurin/nuclear factor of activated T cell (NFAT) pathway^16,17^. Purinergic signaling also plays a critical role in cytosolic Ca^2+^-dependent SC differentiation^15^ and proper myelin formation^18^. Moreover, calcium signaling is also regulated by Cx32 hemichannels^19^ and β-arrestin-mediated signaling^20^ during SC proliferation or differentiation.

Transient receptor potential (TRP) channels are a group of non-selective Ca^2+^-permeable cation channels that are expressed and activated in many cell types and tissues. Most TRP channels localize to the plasma membrane where they act as important mediators of sensory signals and play critical roles in calcium homeostasis. TRP vanilloid 4 (TRPV4), a member of the TRPV channel subfamily, is activated by various stimuli, including mechanical stimulation, moderate heat, and osmolarity, as well as several endogenous (e.g., anandamide, arachidonic acid, and its epoxyeicosatrienoic acid metabolites) and exogenous chemicals^21^. Previous reports showed that TRPV4 is widely expressed and activated in hippocampal neurons^22^ and various glial cells including astrocytes^23^, microglia^24^ and oligodendrocytes^25^ in the central nervous system (CNS). In the PNS, TRPV4 is reported to be expressed in populations of DRG neurons^26^, satellite glial cells^27^, and Müller glial cells^28^. In DRG neurons, TRPV4 mediates nociceptive responses to hypotonic stimuli^26^. However, there are no reports of TRPV4 expression in SCs.

In this study, we characterized the functional expression and physiological significance of TRPV4 channels in SCs. We also demonstrate that TRPV4 is involved in nerve demyelination after sciatic nerve cut injury.

## Results

### Functional TRPV4 is expressed in cultured mouse SCs

**Fig. 1:**
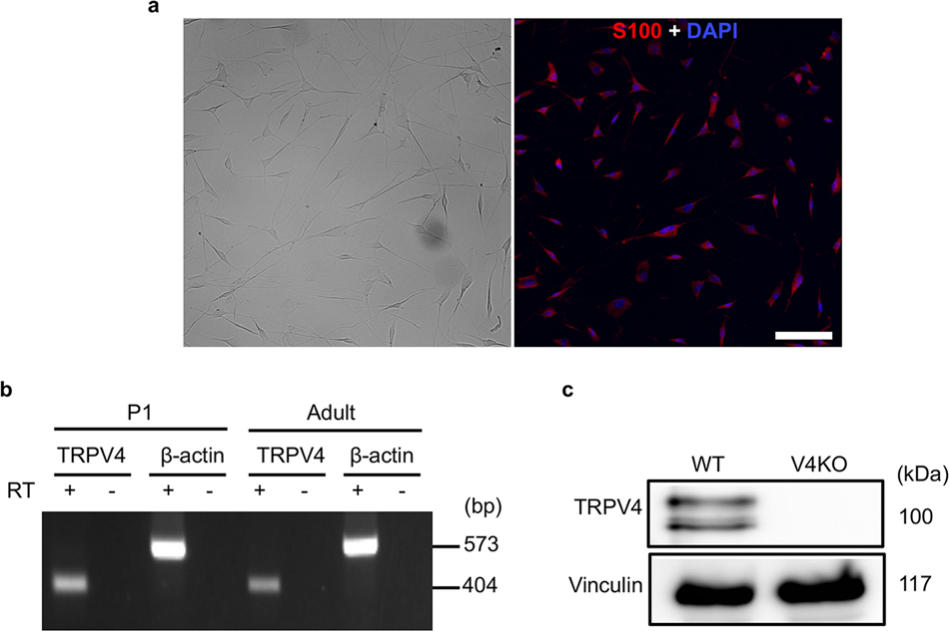
TRPV4 expression in cultured mouse Schwann cells (SCs). **a** Bright-field image of purified primary Schwann cells (SCs) from sciatic nerves of postnatal day 1 (P1) wild-type (WT) mice (left), and double staining of S100 (a specific marker of SCs, red) and DAPI (blue, right). Scale bar: 100 µm. **b** RT-PCR for TRPV4 mRNA in purified primary SCs from P1 and adult mice. RT (+) and (−) represent with and without reverse transcriptase treatment during preparation of cDNA samples, respectively. β-actin mRNA was used as a loading control. The expected sizes of TRPV4 and β-actin are 404 bp and 573 bp, respectively. **c** Western blotting of TRPV4 in purified primary SCs from P1 WT and TRPV4KO (V4KO) mice. Vinculin was used as a loading control. The predicted molecular weights of TRPV4 and vinculin are 100 kDa and 117 kDa, respectively. Two TRPV4 bands were detected in the total cell lysates.

To evaluate the expression of TRPV4 in SCs, we isolated and purified SCs from sciatic nerves from postnatal day 1–3 (P1–3) and adult mice. To isolate SCs from adult mice, we cultured sciatic nerve tissue for 10 days before enzymatic dissociation to facilitate myelin removal (for details see the “Methods”). Accordingly, SCs from P1–3 and adult mice were both non-myelinating SCs. SC purity was confirmed to be >98% by immunostaining for S100, a marker of SCs (Fig. 1a). TRPV4 mRNA was positively amplified from the SCs isolated from pups and adult mice (Fig. 1b). And TRPV4 protein was also detected in cultured wild-type (WT) SCs, but not in SCs from *Trpv4*-deficient (TRPV4KO) mice (Fig. 1c). These results indicated that TRPV4 is expressed in cultured SCs.

**Fig. 2:**
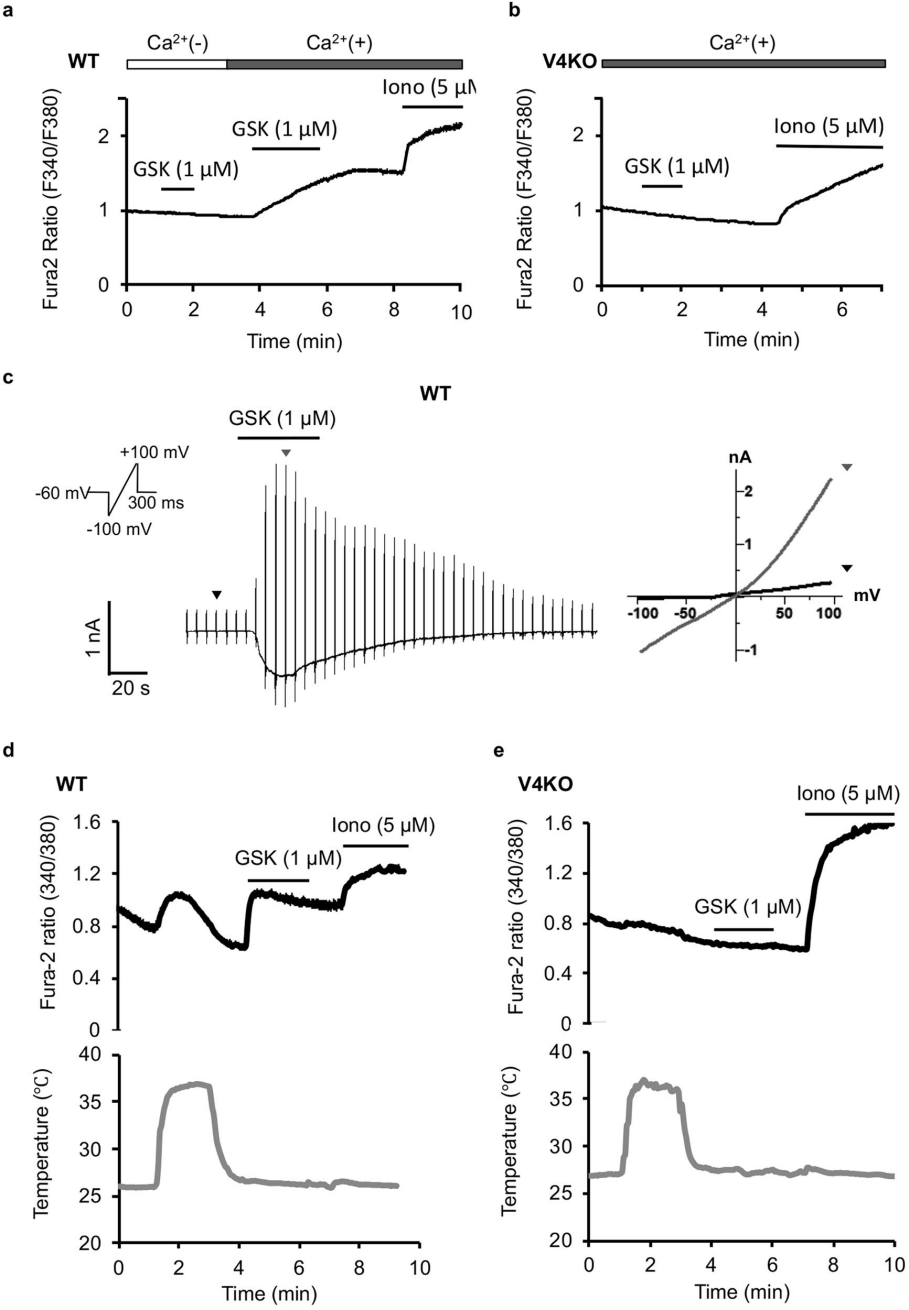
TRPV4 is functionally expressed in cultured mouse SCs. **a**, **b** Mean Fura-2 ratios corresponding to intracellular Ca^2+^ concentrations in primary SCs isolated from adult WT (**a**, *n* = 50 cells) or TRPV4KO mice (**b**, *n* = 121 cells). SCs were stimulated with the TRPV4-selective agonist GSK1016790A (GSK, 1 μM) in the presence or absence of 2 mM extracellular Ca^2+^. Ionomycin (iono, 5 μM) was applied to confirm cell viability. Data are presented as the mean ± SEM. **c** Representative trace of GSK-induced currents in primary SCs from adult WT mice using a whole-cell patch-clamp method (left). Holding potential was −60 mV, and ramp-pulses from −100 to +100 mV (inset) were applied for 300 ms every 5 s. Current–voltage (I–V) curves at the time points indicated by the triangles are shown at right. **d**, **e** Mean Fura-2 ratios in response to temperature elevation up to 37 °C in primary SCs isolated from adult WT (**a**, *n* = 33 cells) or TRPV4KO (**b**, *n* = 51 cells) mice. The lower traces (gray) represent the real-time temperature during calcium imaging. GSK (1 μM) was applied as a positive control for the TRPV4 response. Data are presented as the mean ± SEM.

In addition to this molecular confirmation, further validation of the functional expression of TRPV4 was performed by calcium-imaging and whole-cell patch-clamp recording. In the presence of 2 mM extracellular Ca^2+^ in bath solution, 1 µM of the selective TRPV4 agonist GSK1016790A (GSK) induced an increase in intracellular Ca^2+^ concentrations ([Ca^2+^]_i_) in cultured WT SCs (Fig. 2a), but not in TRPV4KO SCs (Fig. 2b), indicating the functional expression of TRPV4 in SCs. Moreover, the GSK-induced [Ca^2+^]_i_ increase was dependent on extracellular Ca^2+^ in WT SCs (Fig. 2a), suggesting that the [Ca^2+^]_i_ increase is caused by Ca^2+^ influx through TRPV4 on the plasma membrane, and not from intracellular Ca^2+^ stores. TRPV4-mediated responses to GSK (1 µM) were also detected in whole-cell patch-clamp recordings with an outwardly rectifying current–voltage (I–V) relationship (Fig. 2c). Combined, these results demonstrated the functional expression of TRPV4 in cultured mouse SCs.

Since TRPV4 is a member of the TRP channel family, we also checked the expression of several other TRP channels in purified SCs. mRNA for TRPV1, TRPV2, TRPV3, TRPV4, and TRPA1 was detected whereas that for TRPV5 and TRPM3 was not (Supplementary Fig. 1a). Capsaicin, allyl isothiocyanate (AITC), camphor, pregnenolone sulfate (PS) and lysophosphatidylcholine (LPC), agonists of TRPV1, TRPA1, TRPV3, TRPM3 and TRPV2, respectively, evoked either no or a negligible increase (for LPC) in [Ca^2+^]_i_ (Supplementary Fig. 1b–d), suggesting that SCs do not express functional TRPV1, TRPV2, TRPV3 and TRPA1 even though mRNA expression occurs. Given the report showing functional expression of TRPA1 in mouse SCs^22^, we also tested high concentrations of AITC (1 mM), which did not increase [Ca^2+^]_i_, whereas low concentrations of GSK (50 nM) did (Supplementary Fig. 1e). Taken together, these results suggest that TRPV4 is one of the primary thermosensitive TRP channels that are functionally expressed in SCs.

### TRPV4 is activated at normal body temperature in mouse SCs

As a TRP channel that is sensitive to warm temperatures (>30 °C), TRPV4 should be constitutively activated in SCs at normal body temperature in the body. Here we measured temperature-evoked [Ca^2+^]_i_ changes in cultured SCs. Temperature elevation to 37 °C induced an increase in [Ca^2+^]_i_ similar to that seen after application of 1 μM GSK in WT (Fig. 2d), but not TRPV4KO SCs (Fig. 2e). These results suggested that TRPV4 is constitutively active in SCs in the PNS under physiological conditions, which is consistent with previous reports for other cell types^29,30^.

### TRPV4 does not affect normal myelin development in the peripheral nervous system

We next assessed the physiological function of TRPV4 in SCs. Given the key role of SCs in myelin formation, we first examined whether TRPV4 is involved in normal myelin development of SCs in the PNS in WT and TRPV4KO mice. The motor functions and sensory inputs as well as overall general health factors (e.g., body weight, body temperature and circadian rhythm) of TRPV4KO mice were all similar to those of WT littermates as previously reported^30^.

**Fig. 3:**
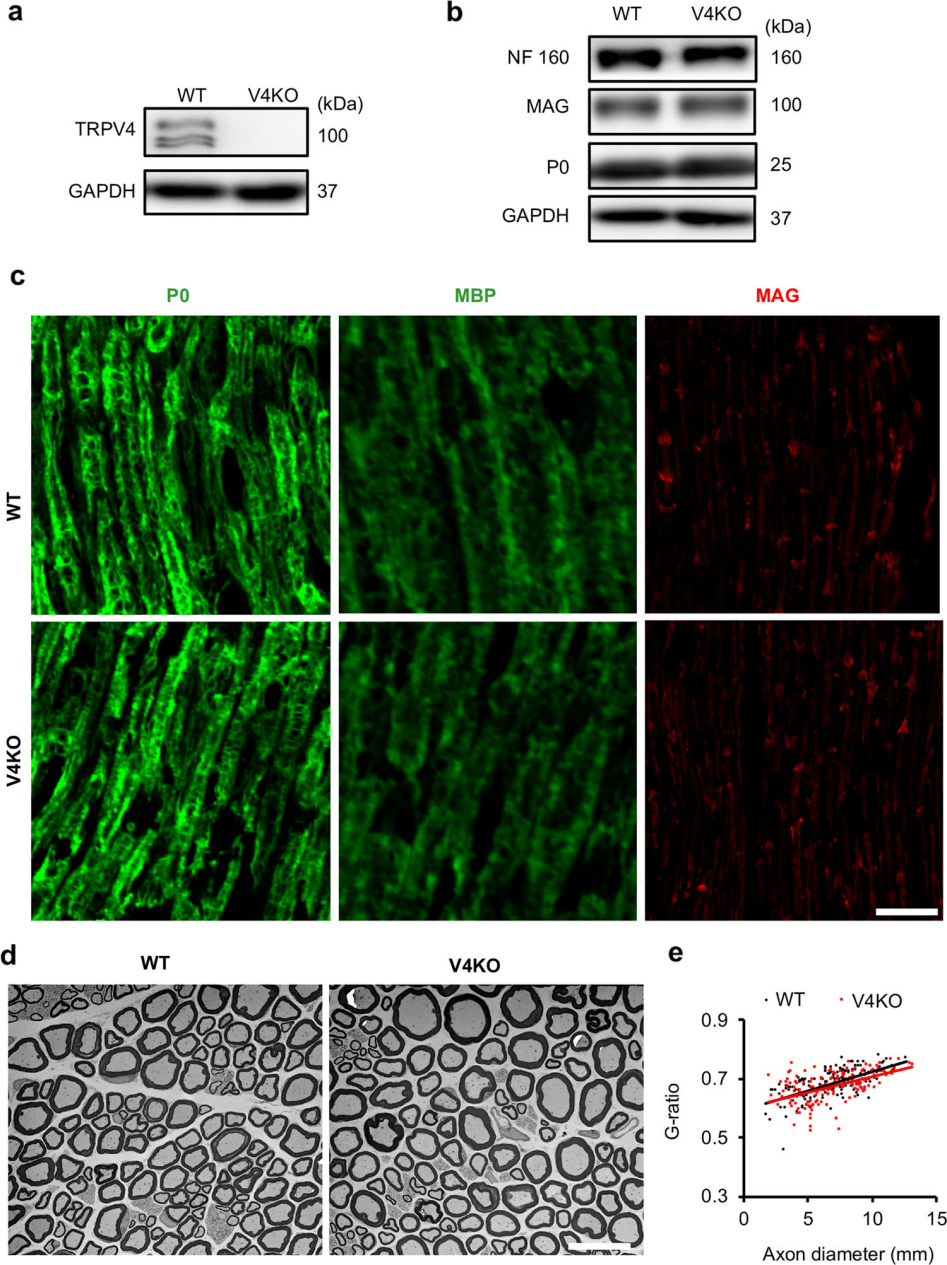
TRPV4KO mice develop normal myelin in adulthood. **a** Western blotting of TRPV4 in sciatic nerve tissues from 15-week-old WT and TRPV4KO mice. GAPDH was used as a loading control. The predicted molecular weight of GAPDH is 37 kDa. **b** Western blotting of neurofilament (NF) 160, myelin-associated glycoprotein (MAG) and myelin protein zero (P0) in sciatic nerves from 15-week-old WT and TRPV4KO littermates. The predicted molecular weights of NF 160, MAG and P0 are 160 kDa, 100 kDa and 25 kDa, respectively. **c** Representative immunostaining images of P0 (green), myelin basic protein (MBP, green) and MAG (red) expression in longitudinal sections of sciatic nerves from 15-week-old WT (top) and TRPV4KO (bottom) mice. Scale bar: 40 µm. **d** Representative transmission electron microscopy (TEM) images of transverse sections of sciatic nerves from 15-week-old WT and TRPV4KO mice. Scale bar: 20 µm. **e** Scatter plot of g-ratios corresponding to individually measured axons from 15-week-old WT (black) and TRPV4KO (red) mice (*n* = 162 axons from 3 WT mice, and *n* = 150 from 3 TRPV4KO mice). Trend lines were generated by multiple linear regression analysis.

Myelin lamellae are divided into two domains, non-compact and compact. Non-compact myelin is involved in SC-axon or SC-SC interactions, whereas compact myelin contributes to formation of highly organized myelin lamellae in electrically insulated axons. Myelin-associated glycoprotein (MAG) is expressed in non-compact myelin that is crucial for myelin and axonal maintenance. Meanwhile, myelin protein zero (P0) and myelin basic protein (MBP) localize in compact myelin, which is an important component of the myelin sheath in peripheral nerves^8,31^. Although we confirmed TRPV4 expression in sciatic nerves from 15-week-old WT mice by western blot (Fig. 3a), we saw no differences in expression levels of P0, MBP or MAG in sciatic nerves from WT and TRPV4KO littermates (Fig. 3b, c). We also found similar protein levels of the axonal marker Neurofilament (NF) 160 in WT and TRPV4KO sciatic nerves (Fig. 3b). Furthermore, we observed no difference in myelin structure between 15-week-old WT and TRPV4KO mice by transmission electron microscopy (TEM; Fig. 3d). The axon diameters and myelin thickness were similar between the two genotypes, and this similarity was further confirmed by the similar g-ratios (the ratio of the axonal diameter to the diameter of the total axon, including the myelin, Fig. 3e).

Given that myelination occurs over an extended period during the first three weeks of postnatal life in mice^1^, we compared MAG and P0 protein levels in sciatic nerves from WT and TRPV4KO littermates on postnatal day 7 (P7), postnatal day 15 (P15), postnatal day 21 (P21) and 8 weeks (Supplementary Fig. 2). MAG and P0 protein levels were comparable between WT and TRPV4KO siblings at all developmental stages. Taken together, our results suggested that loss of TRPV4 does not affect normal myelin development in the PNS in mice.

### TRPV4 expression is increased in SCs during sciatic nerve injury

**Fig. 4:**
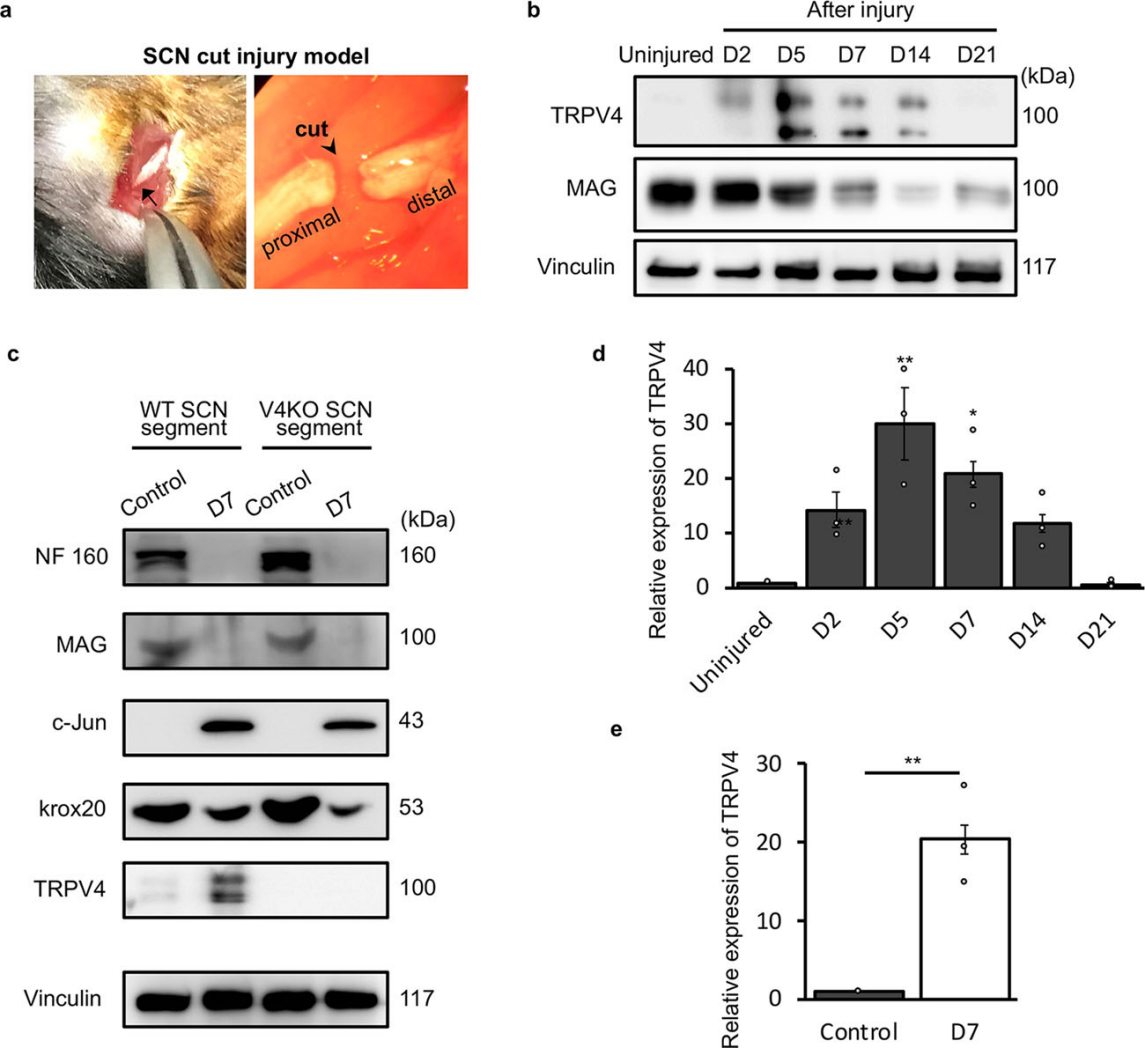
TRPV4 expression is increased in demyelinating sciatic nerves after injury. **a** Sciatic nerve cut injury model in mice. The sciatic nerve was exposed and cut (arrowhead) at the notch site (arrow) in 15-week-old mice. Distal stumps were collected for subsequent experiments. **b** Representative western blots of TRPV4 and MAG proteins in WT sciatic nerves between 2 days (D2) and 3 weeks (D21) after injury. Vinculin was used as a loading control. **c** Representative western blots of NF 160, MAG, c-Jun, krox20 and TRPV4 proteins in cultured sciatic nerve (SCN) segments from WT and TRPV4KO mice. ‘Control’ represents uncultured sciatic nerve segments; ‘D7’ represents sciatic nerve segments cultured for 7 days. The predicted molecular weights of c-Jun and krox20 are 43 kDa and 53 kDa, respectively. **d** TRPV4 protein expression levels normalized relative to vinculin shown in (**b**). Data are presented as the mean ± SEM. One-way ANOVA followed by a Bonferroni post-hoc test was used for comparison. **P < 0.01,*P < 0.05 compared to the uninjured group. **e** TRPV4 protein expression levels normalized relative to vinculin shown in (**c**). Data are presented as the mean ± SEM. A two-tailed *t*-test was used for comparison. **P < 0.01 compared to the uninjured group. *N* = 3 independent experiments.

SCs are known to exhibit high plasticity in response to environmental stimuli. And TRPV4 can be sensitized by inflammatory responses^32^ or abnormal mechanical pressures produced by volume changes in interstitial fluid^33^ during injury. Thus, we investigated whether TRPV4 affects SC differentiation under pathological conditions, such as sciatic nerve cut injury. Sciatic nerves in 15-week-old WT mice were cut at the notch region (Fig. 4a), resulting in Wallerian degeneration of the distal nerve stumps. NF 160 expression was completely absent by 5 days after injury, and regeneration was initiated 14 days after injury. P0 and MAG expression began to decrease shortly after injury, and the lowest levels were seen 14 days after injury when regeneration began (Supplementary Fig. 3). Interestingly, TRPV4 levels largely increased with sciatic nerve demyelination between 2 and 14 days after injury, and returned to basal levels during sciatic nerve remyelination at 21 days after injury (Fig. 4b, d).

To explore whether the increased TRPV4 protein levels during the demyelinating phase of sciatic nerve injury arise from SCs, we attempted to assess TRPV4 protein levels in SCs after nerve injury. However, in double immunostaining of TRPV4 and S100β, the TRPV4 antibody had high nonspecific immunoreactivity in long-term (>4 h) PFA-fixed tissues. To overcome this issue, we performed an in vitro experiment of sciatic nerve cut injury to exclude contamination by TRPV4 expressed in other cell types. Culture of short nerve segments in vitro causes progressive myelin breakdown, similar to the changes seen in vivo. However, these processes are mainly performed by SCs since DRG neurons and invading immune cells are absent^7^. After 7 days of in vitro culture, short nerve segments underwent Wallerian degeneration, which was consistent with processes observed in vivo. Both NF 160 and MAG expression were nearly absent (Fig. 4c). Expression levels of c-Jun, a well-known regulator of demyelination and repair were elevated, whereas expression of Krox20, a positive regulator of myelination, was diminished. Importantly, nerve segments cultured in vitro displayed an ~20-fold increase in TRPV4 protein levels relative to freshly isolated nerve segments (Fig. 4c, e), indicating that TRPV4 expression is increased in SCs during the demyelinating phase after nerve injury.

### Increased TRPV4 expression in SCs after injury is due to an increase in numbers of non-myelinating SCs

**Fig. 5:**
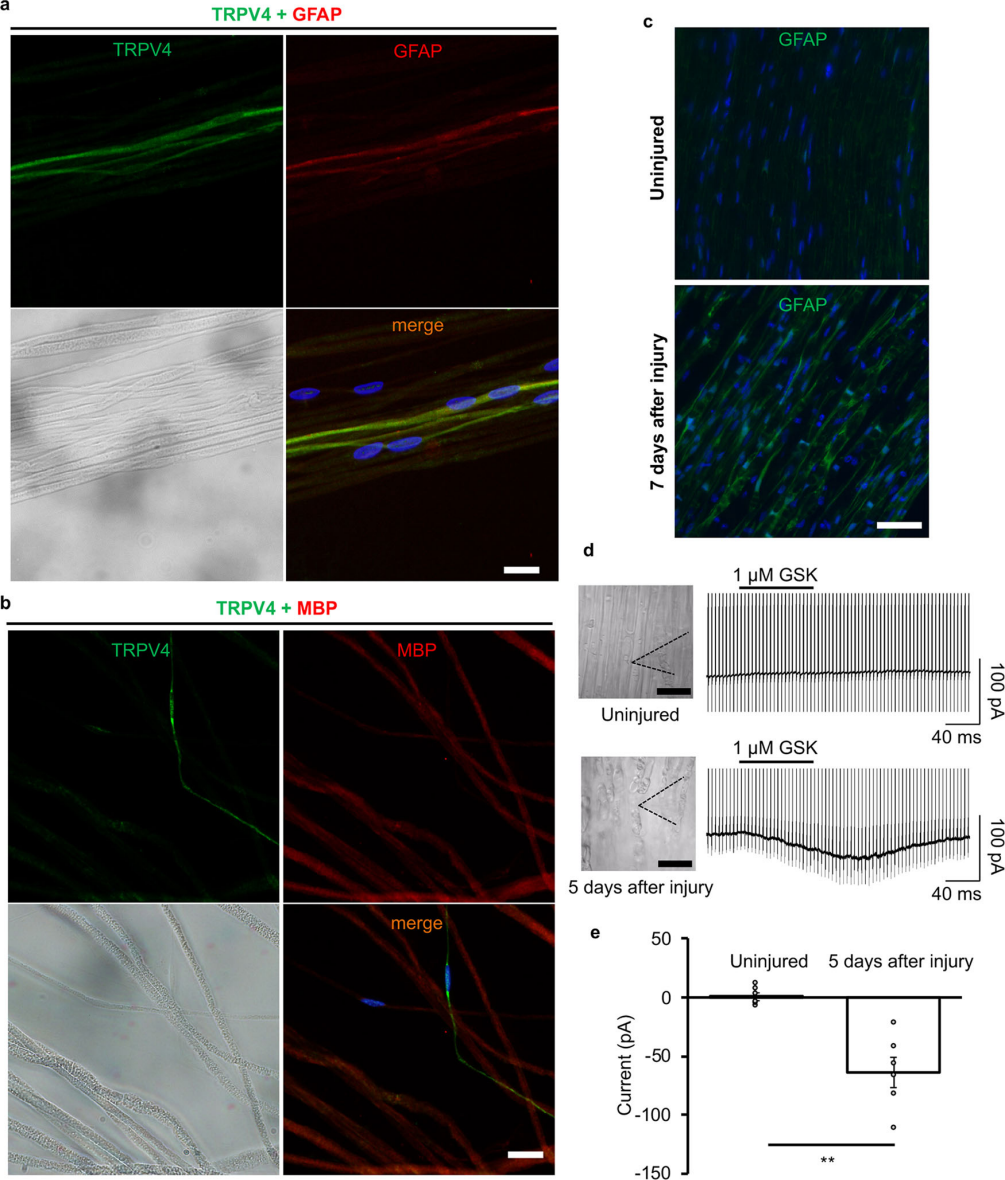
TRPV4 expression in non-myelinating SCs is increased in the demyelinating phase after injury. **a**, **b** Immunostaining images of TRPV4 (green), DAPI (blue), and GFAP (red, **a**) or MBP (red, **b**) in nerve fibers teased apart from the sciatic nerve of 15-week-old WT mice. The left lower panels show bright-field images. Scale bars: 10 µm. **c** Immunostaining images of GFAP (green) and DAPI (blue) in longitudinal sciatic nerve sections taken from uninjured (upper) or injured (lower) 15-week-old WT mice. Scale bar: 50 µm. **d** Representative photographs of ex-vivo patch-clamp recording on a myelin sheath of a SC from distal sciatic nerve bundles taken from uninjured (left upper) or injured (left lower) 15-week-old WT mice. Black dotted lines trace the pipette outline (left). Scale bars: 10 μm. Representative traces of 1 μM GSK-induced currents from the recording shown at left are shown (right). The holding potential was −60 mV, and ramp-pulses from −100 to +100 mV were applied for 300 ms every 5 s. **e** Comparison of inward currents at −60 mV induced by 1 μM GSK in myelin sheaths of SCs from uninjured or injured WT mice shown in (**d**). Data are shown as the mean ± SEM (*n* = 6 cells each group). **P < 0.01.

To further understand how TRPV4 protein expression is regulated, we first analyzed expression patterns of TRPV4 in teased sciatic nerves. Sciatic nerves were isolated and individual fibers were carefully teased apart under a dissection microscope. TRPV4 immunoreactivity was positive in WT teased sciatic nerves, but not in TRPV4KO teased nerves, which confirmed the antibody specificity (Supplementary Fig. 4). Glial fibrillary acidic protein (GFAP) was used to label non-myelinating SCs^34^, whereas MBP was used to label myelinating SCs. Double immunostaining of uninjured teased sciatic nerves from WT mice revealed that TRPV4 is expressed exclusively in non-myelinating SCs (GFAP-positive cells, Fig. 5a) and not in myelinating SCs (MBP-positive cells) (Fig. 5b). Moreover, GFAP expression was increased in the distal stump 7 days after sciatic nerve injury (Fig. 5c), indicating that the number of non-myelinating SCs increased after injury. These results suggested that the observed increase in TRPV4 protein is likely due to this increase in non-myelinating SCs.

To further examine changes in TRPV4 expression in SCs during the demyelinating phase, we performed ex-vivo whole-cell patch-clamp recording of the myelin sheath of SCs from uninjured or injured distal sciatic nerves. No response was induced by 1 μM GSK in SCs from uninjured sciatic nerves, but GSK-evoked inward currents (−63.5 ± 12.9 pA; Fig. 5d, e) were observed in SCs from injured sciatic nerves, indicating that TRPV4 expression in SCs is induced by nerve demyelination after injury. Together, these experiments provide evidence that TRPV4 is exclusively expressed in non-myelinating rather than myelinating SCs, while distal nerves are gradually demyelinated after injury, leading to increased expression of TRPV4 protein in SCs.

### TRPV4 deletion inhibits demyelination processes that further inhibit remyelination in SCs after sciatic nerve injury

**Fig. 6:**
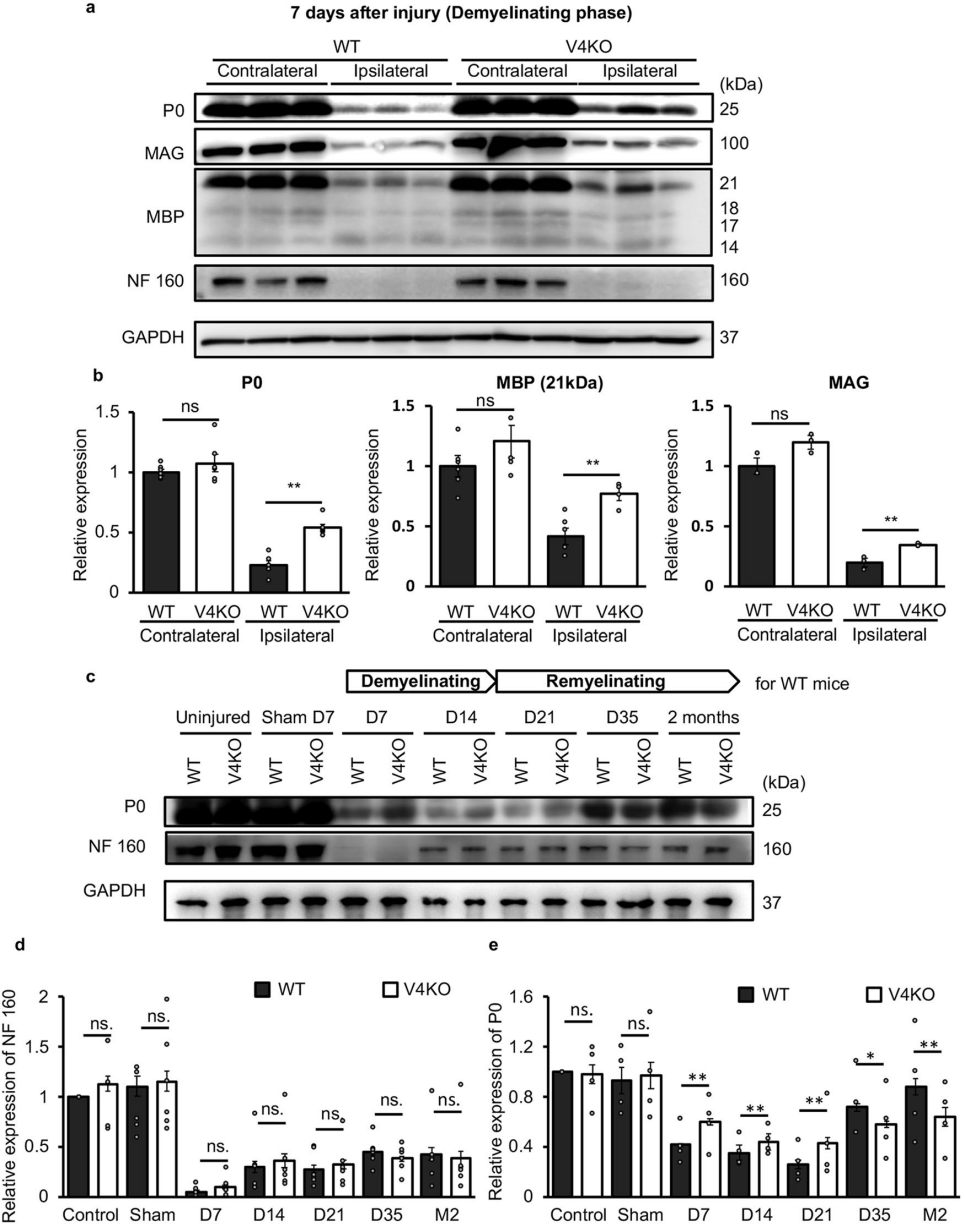
TRPV4 deletion alters the level of myelin-related proteins in demyelination and remyelination processes during sciatic nerve injury. **a** Representative western blot images of P0, MAG, MBP, and NF 160 proteins in contralateral and ipsilateral distal sciatic nerves from 15-week-old WT and TRPV4KO mice at 7 days after injury during the demyelinating phase. **b** Expression levels of proteins shown in (a) normalized to GAPDH. Data are presented as mean ± SEM. Each group had 6 mice and experiments were repeated 3 times. A two-tailed *t*-test was used for comparison. **P < 0.01, ns. = not significant. **c** Representative western blots of P0 and NF160 protein in distal sciatic nerves between 7 days (D7) and 2 months after injury in 15-week-old WT and TRPV4KO mice. “Demyelinating” phase and “Remyelinating” phase indicate from 7 days to 14 days and from 21 days to 2 months after injury for WT mice, respectively. “Uninjured” indicates expression levels in sciatic nerves without injury. “Sham” indicates expression levels in sciatic nerves that were exposed, but not cut. **d**, **e** Expression levels of NF 160 (**d**) and P0 (**e**) proteins shown in (**c**) normalized to GAPDH. Data are presented as the mean ± SEM. *n* = 3 independent experiments. A two-tailed *t*-test was used for comparison. **P < 0.01, ns. = not significant.

We propose that distal nerve demyelination induced by sciatic nerve injury could be affected by increased TRPV4 expression in SCs. To examine this possibility, we used the sciatic nerve cut injury model in both WT and TRPV4KO littermates, and evaluated axon- and myelin-related proteins 7 days after injury by western blot. Levels of NF 160, P0, MAG, and MBP were all decreased in both genotypes after injury, indicating axon degeneration and SC demyelination as we described (Supplementary Fig. 3). However, compared to WT mice, TRPV4KO mice showed significantly higher levels of P0, MAG, and MBP proteins in the ipsilateral distal stumps, but not in the contralateral side (Fig. 6a, b). These results suggest partial blockade of demyelination processes in TRPV4KO mice.

Incomplete demyelination is expected to inhibit nerve remyelination during the recovery stage of sciatic nerve injury-induced Wallerian degeneration^35^. We thus further examined the time course of NF 160 and P0 expression from 7 days to 2 months after injury and found that levels of these two proteins first decreased, and then increased after injury in both WT and TRPV4KO mice. There were no clear differences in NF 160 expression between the two genotypes (Fig. 6c, d), indicating that axon degeneration and regeneration were not affected by TRPV4 deficiency. On the other hand, compared with WT mice, P0 protein levels were significantly higher in TRPV4KO mice 1–3 weeks after injury during the demyelination process, but were significantly lower in TRPV4KO mice 5 weeks to 2 months after injury when the remyelination process is ongoing (Fig. 6c, e). This result suggested that blockade of demyelination in TRPV4KO mice, in turn, hindered remyelination during the recovery process of sciatic nerve injury-induced Wallerian degeneration.

### Lack of TRPV4 delays functional recovery and remyelination of injured sciatic nerves

**Fig. 7:**
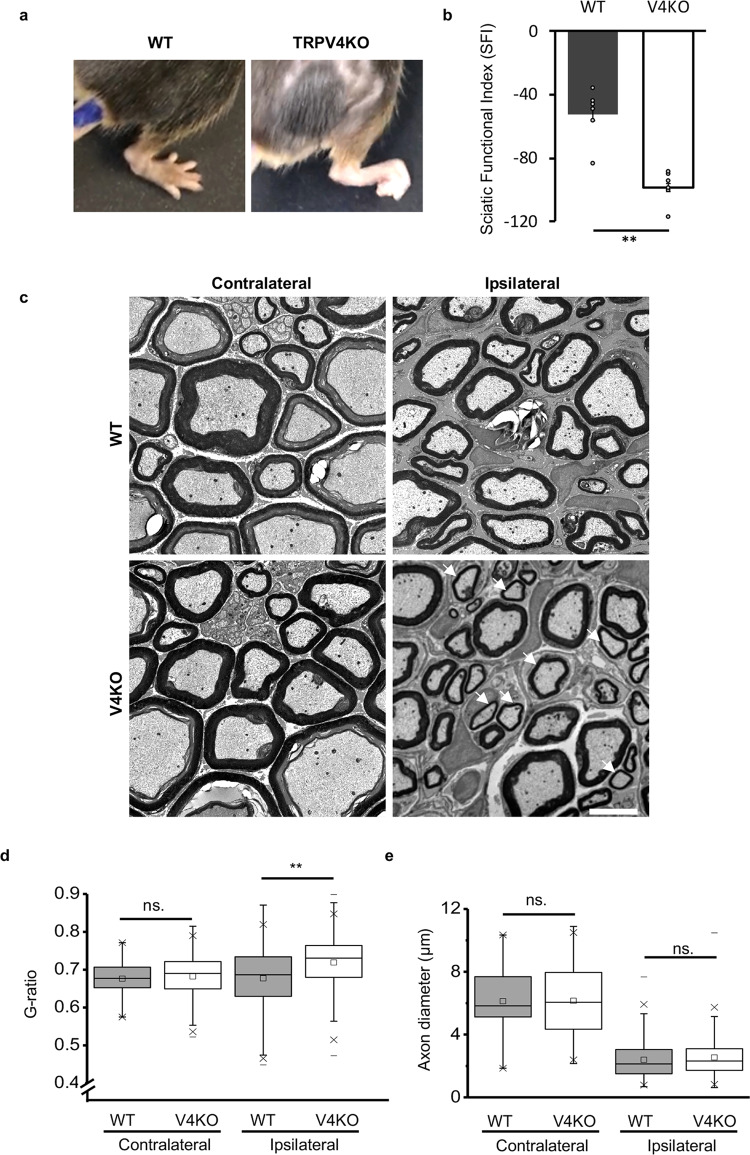
Loss of TRPV4 impairs functional recovery and remyelination of sciatic nerves 2 months after injury. **a** Representative appearance of ipsilateral hind paws of WT and TRPV4KO mice 2 months after injury. Paws of all the WT mice exhibited a normal spread, whereas 4/6 TRPV4KO mice showed severe contracture. **b** Sciatic nerve function in WT and TRPV4KO mice (*n* = 6 mice each) was evaluated with the sciatic functional index (SFI). SFI values were calculated using the formula: ${\mathrm{Sciatic}}\,{\mathrm{functional}}\,{\mathrm{index}}\,\left( {{\mathrm{SFI}}} \right) = 118.91 \ast \frac{{{\mathrm{ETS}} - {\mathrm{NTS}}}}{{{\mathrm{NTS}}}} - 51.25 \ast \frac{{{\mathrm{EPL}} - {\mathrm{NPL}}}}{{{\mathrm{NPL}}}} - 7.57$ Where ETS: toe spread (distance between the first and fifth toe) of the ipsilateral hind paw; NTS: toe spread of the contralateral hind paw; EPL: paw length (distance from the heel to the third toe) of the ipsilateral hind paw; NTS: paw length of the contralateral hind paw. Data are presented as the mean ± SEM (*n* = 6 each group). **p < 0.01. **c** Representative TEM images of transverse sections of distal sciatic nerves from WT contralateral (top left), WT ipsilateral (top right), TRPV4KO contralateral (lower left), and TRPV4KO ipsilateral (lower right) 2 months after injury. White arrows indicate the apparent thinning of myelin sheaths in the ipsilateral sciatic nerves from TRPV4KO mice relative to WT mice. Scale bar: 5 µm. **d** G-ratios of contralateral (*n* = 70 axons for WT, and *n* = 140 for TRPV4KO mice) and ipsilateral (*n* = 707 axons from WT mice, and *n* = 728 from TRPV4KO mice) distal sciatic nerves in WT (*n* = 3) and TRPV4KO (*n* = 3) mice 2 months after injury. Data are presented as the mean ± SEM. A two-tailed *t*-test was used for comparison. **P < 0.01, ns. = not significant. **e** Comparison of axon diameters in contralateral and ipsilateral distal sciatic nerves shown in (**d**). Data are presented as the mean ± SEM. A two-tailed *t*-test was used for comparison. ns. = not significant.

To provide further support for the involvement of TRPV4 in the nerve recovery process, we examined the phenotype of WT and TRPV4KO mice 2 months after sciatic nerve injury. When walking in an open field area, the hind paws on the injured side in WT mice were spread wider than those of the TRPV4KO mice and most (4/6) TRPV4KO mice showed severe foot contracture with the toes curled under the paw (Movie S1 and S2 and Fig. 7a). To assess functional recovery of the injured sciatic nerve, we used walking track analysis to quantify the walking gaits of these mice. Sciatic functional index (SFI) values were calculated as previously described^36,37^. The SFI values were significantly smaller for TRPV4KO mice than for WT mice (Fig. 7b; −52.7 ± 3.9 for WT mice vs. −98.3 ± 2.5 for TRPV4KO mice; *p* = 0.0002), indicating that the absence of TRPV4 impairs the functional recovery of sciatic nerves at 2 months after injury.

To further evaluate the structure of remyelinated sciatic nerves after injury, the distal stumps from these mice were collected and analyzed by TEM. The diameter in ipsilateral sections of the regenerated axons appeared to be smaller compared to the contralateral sides for both genotypes, although no differences in the contralateral sections were observed between WT and TRPV4KO mice (Fig. 7c, left). Importantly, TRPV4KO mice appeared to have thinner myelin sheaths compared to WT mice (Fig. 7c, right). Indeed, the average g-ratios were significantly larger in TRPV4KO mice than in WT mice (0.718 ± 0.003 for TRPV4KO vs. 0.677 ± 0.003 for WT mice; *p* < 0.0001; Fig. 7d), indicating that the regenerated myelin was in fact thinner in TRPV4KO mice than in WT mice. The average axon diameters showed no clear differences between WT or TRPV4KO mice (2.188 ± 0.038 in WT vs. 2.291 ± 0.037 in TRPV4KO mice; *p* = 0.053) and the axon diameters were significantly smaller on the ipsilateral sides in both genotypes (Fig. 7c, e). Moreover, scatter plots showed apparently higher g-ratios for all axon sizes in TRPV4KO mice 2 months after injury, suggesting that the thinner myelin in TRPV4KO mice is independent of axon size (Supplementary Fig. 5).

**Fig. 8:**
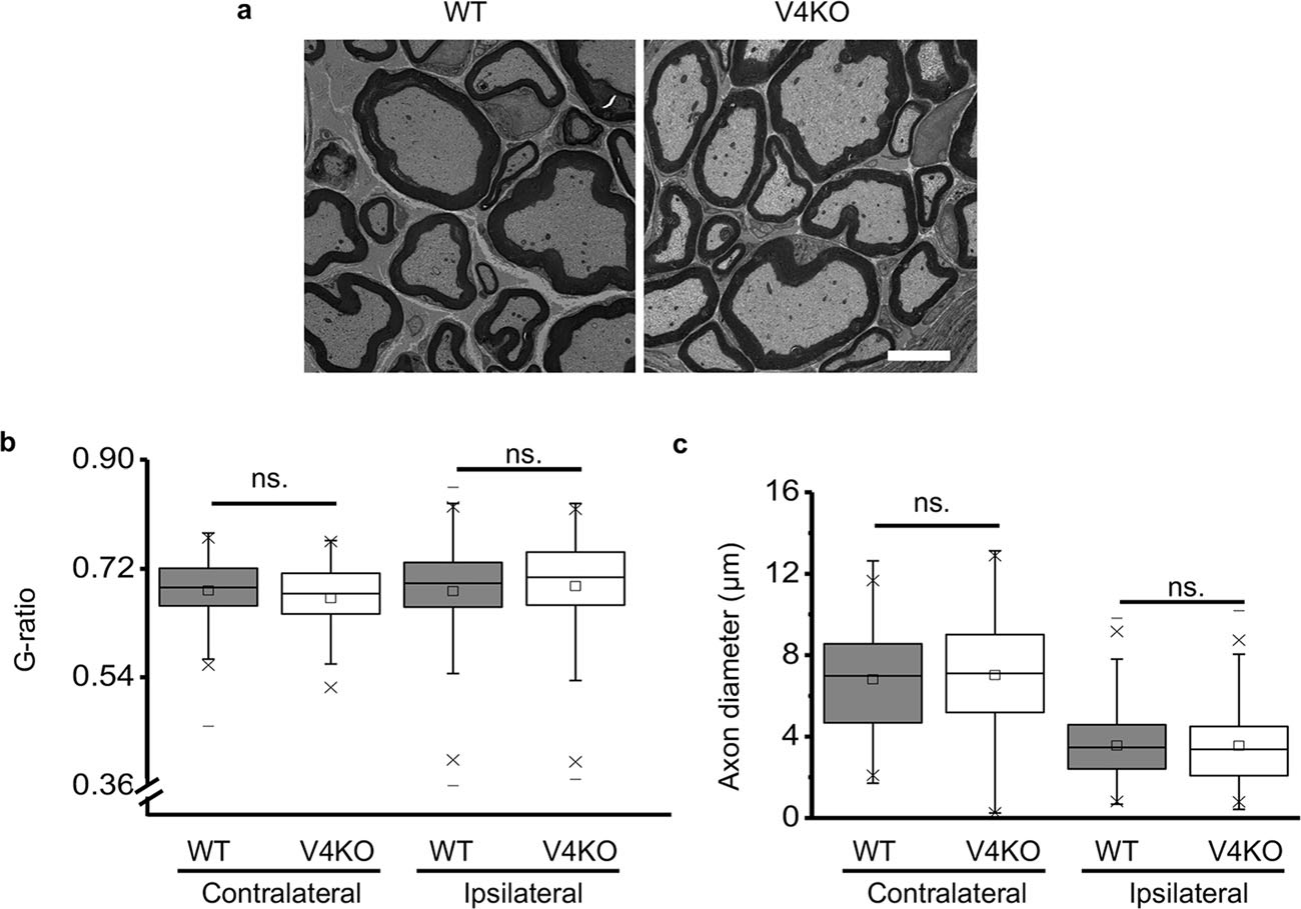
Restoration of TRPV4-dependent sciatic nerve remyelination 6 months after injury. **a** Representative TEM images of transversely sectioned distal sciatic nerves from WT ipsilateral (left) and TRPV4KO ipsilateral (right) 6 months after injury. Scale bar: 5 µm. **b** G-ratios of ipsilateral distal sciatic nerves in WT (*n* = 231 axons from 4 mice) and TRPV4KO (*n* = 283 axons from 4 mice) mice 6 months after injury. Data are presented as the mean ± SEM. A two-tailed *t*-test was used for comparison. ns. = not significant. **c** Axon diameters of contralateral and ipsilateral distal sciatic nerves shown in (**b**). Data are presented as the mean ± SEM. A two-tailed *t*-test was used for comparison. ns. = not significant.

When we examined the structure of remyelinated sciatic nerves at 6 months after injury, the regenerated sciatic nerves were similar between the two genotypes (Fig. 8a). This observation was supported by the calculated g-ratios and axon diameters, although axon diameters were still significantly smaller in the ipsilateral stumps for both genotypes (Fig. 8b, c), suggesting that the role of TRPV4 in SC could be compensated for in the chronic stage. Taken together, these results demonstrate the lack of TRPV4 delays sciatic nerve remyelination and functional recovery following sciatic nerve injury-induced Wallerian degeneration.

## Discussion

**Fig. 9:**
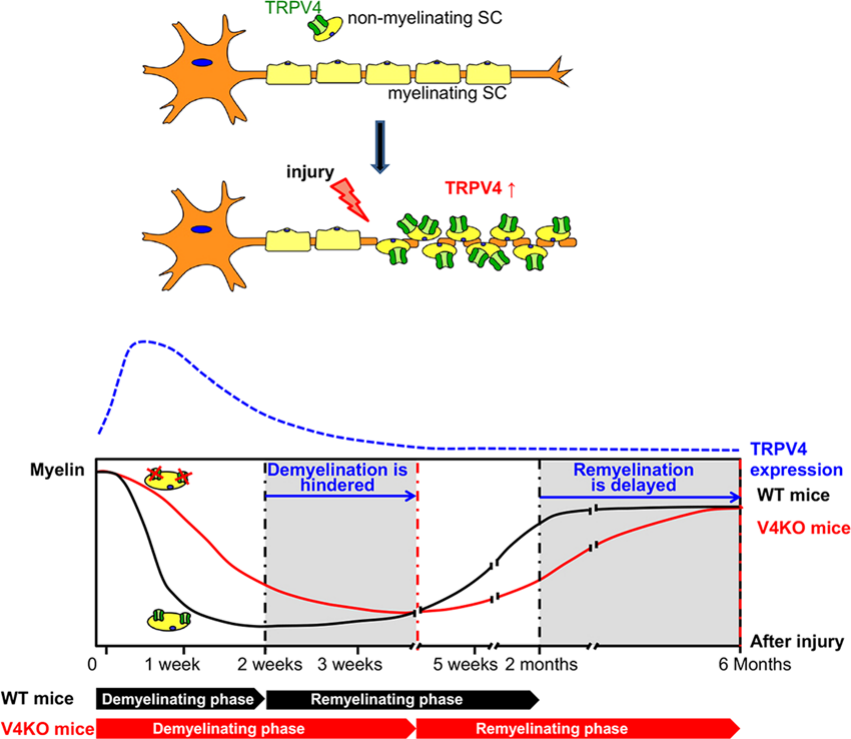
Schematic presentation of TRPV4 function in SCs after sciatic nerve cut injury. TRPV4 is expressed in non-myelinating SCs but not in myelinating SCs. After sciatic nerve cut injury, SCs from the distal nerve are gradually demyelinated causing an increase of non-myelinating SCs that, in turn, leads to an increase in TRPV4 protein expression. TRPV4 is activated under physiological conditions and enhances SC demyelination. A lack of TRPV4 hinders SC demyelination 1–2 weeks after injury. This incomplete demyelination delays remyelination of sciatic nerves in TRPV4KO mice 2 months to 6 months after injury. The dashed blue line indicates changes in TRPV4 levels in distal nerves corresponding to days after injury; black and red solid lines indicate changes in myelin-related protein levels corresponding to the number of days after injury to distal nerves from WT and TRPV4KO mice, respectively.

In this study, we demonstrated and characterized the functional expression of TRPV4 in SCs in peripheral nerves. To the best of our knowledge, this is the first to describe the function and expression of TRPV4 in SCs. Our results suggested a role for TRPV4 in the sciatic nerve cut injury model (Fig. 9). TRPV4 is exclusively expressed in non-myelinating rather than myelinating SCs. Following sciatic nerve cut injury, distal nerves are gradually demyelinated, resulting in an increase in the levels of TRPV4 proteins in SCs. TRPV4 channels are activated in non-myelinating SCs at body temperature and nerve demyelination is enhanced. The absence of TRPV4 hinders nerve demyelination, which in turn delays the functional recovery and remyelination of sciatic nerves after injury. However, whether TRPV4 has a direct role in remyelination processes cannot be definitively determined based on our current results, and thus further investigations are needed to examine TRPV4 function during remyelination in greater detail.

Our study provides both molecular and physiological evidence showing that TRPV4 is functionally expressed in SCs. We observed high expression of TRPV4 levels in cultured SCs (Fig. 1) and low expression in naïve sciatic nerves (Figs. 6c, 7). In culture, SCs from both neonatal and adult mice are non-myelinating, whereas in vivo a large proportion of SCs have a myelinating phenotype in sciatic nerves. This outcome could further support our finding that TRPV4 expression is upregulated in non-myelinating SCs, which is consistent with the immunostaining result in teased sciatic nerves (Fig. 5). However, future studies are needed to characterize the sub-cellular localization of TRPV4 protein in SCs.

Several lines of evidence show that TRPV4 expression dynamically changes in SCs during cell type conversions after sciatic nerve injury (Figs. 4, 5). TRPV4 expression levels were increased rapidly in the demyelinating phase after sciatic nerve injury, even in the absence of DRG neurons and invading immune cells (Fig. 4). Indeed, the increase in TRPV4 expression occurred earlier than SC demyelination (Fig. 4b), suggesting that TRPV4 homeostasis in myelinating and non-myelinating SCs is necessary for maintenance of SC plasticity. Peripheral nerve injury could upregulate TRPV4 levels in myelinating SCs to induce conversion of cell types into those having demyelinating phenotypes. High expression levels of TRPV4 in non-myelinating SCs could in turn accelerate SCs remyelination, resulting in downregulation of TRPV4 expression in remyelinating SCs. Deletion of TRPV4 in mice indeed hindered the demyelinating phase after nerve injury which subsequently delayed remyelination and functional recovery of sciatic nerves (Figs. 6, 7).

Previous studies suggested that Ca^2+^ signaling is an important mediator of SC differentiation during myelin development and remyelination following injury^14–18^. Notably, we showed that TRPV4 is not involved in SC differentiation during myelin development (Fig. 3 and Supplementary Fig. 2), but is important for demyelination after nerve injury (Figs. 6, 7). This role may be because TRPV4 is exclusively expressed in non-myelinating SCs that surround multiple small-caliber axons in normal peripheral nerves. The expression level and activity of TRPV4 is very low in these cells in normal peripheral nerve tissues. However, non-myelinating SCs play key roles in Wallerian degeneration following peripheral nerve injury. As we observed here, TRPV4 expression is rapidly increased in SCs upon injury followed by nerve demyelination. In addition to the basal activation of TRPV4 in SCs at physiological body temperature, TRPV4 activity may also be increased by local inflammatory responses (increased tissue temperature and reduced pH^32^) or abnormal mechanical pressures that due to volume changes in interstitial fluid^33^ after injury. Arachidonic acid and its metabolites that are involved in modulating neuronal function and survival during Wallerian degeneration^38^ are also well-known endogenous agonists of TRPV4. Thus, both TRPV4 expression and activity may be increased in SCs after nerve injury and could in turn activate signaling pathways such as the calcineurin-NFAT pathway or a pathway that coordinates with a purinergic signaling pathway to promote demyelination. Further investigations are required to understand these mechanisms in greater detail.

We cannot exclude the possibility that TRPV4 plays a role in functional recovery of other cell types (e.g., DRG neurons) since knockout of TRPV4 expression in our TRPV4KO mice was global, rather than specific to one cell type. Previous reports suggested that TRPV4 is expressed and functions in a population of DRG neurons that could mediate nociceptive responses to hypotonic stimuli or mechanical pressure^26,39,40^. Moreover, TRPV4 was shown to regulate NGF- or cAMP-induced neurite outgrowth in peripheral nerves^41^. In our study, we did not examine sensory functions in TRPV4KO mice since motor function was seriously impaired in the sciatic nerve cut injury model. Instead, we focused on the recovery of myelinated axons. Our results suggest that TRPV4 is not involved in axon degeneration and regeneration following sciatic nerve cut-induced Wallerian degeneration (Fig. 6). This finding might be due to the low expression levels of TRPV4 in injured axon terminals, since axons are degenerated during the demyelinating phase. Cytokines or molecular regulators secreted from the injury site thus may fail to sensitize TRPV4 channels in the cell bodies of DRG neurons that are distant from the injury site, whereas the basal activity of TRPV4 is likely not sufficient to affect axon extension or regeneration during the recovery process.

We used a sciatic nerve cut injury model to mimic neurotmesis in peripheral nerves, in which axons, connective sheaths and basal lamina tubes are interrupted. Among nerve damage schemes in rodents, the sciatic nerve cut injury model is the most serious nerve injury and requires over 2 months for remyelination and over 6 months for functional recovery of sciatic nerves. Even after 6 months, the recovery is generally poor and the reformed axon and myelin sheaths are thinner than in uninjured nerves. In humans with peripheral nerve injury, almost all patients recovered sensory function, but motor recovery is often limited even after multiple surgical interventions^42^. The results of this study suggest that local activation of TRPV4 could be an attractive alternative therapeutic intervention after peripheral nerve injury, although there are a lot of reports showing that gain-of-function mutation of TRPV4 causes peripheral neuropathies in humans^43–46^.

## Methods

### Mice

15-week-old C57BL/6NCr male and female mice were used. Mice were kept under a 12-h light–dark cycle, at 24 °C with unlimited food and water. TRPV4-deficient (TRPV4KO) mice were maintained on a C57BL6/NCr background^47^. Walking track analysis and electron micrograph experiments were conducted with WT and TRPV4KO male littermates. All animal care and experimental procedures were approved by the institutional Animal Care and Use Committee of the National Institute of Natural Sciences, and performed according to the National Institutes of Health and National Institute for Physiological Sciences guidelines.

### Chemicals

GSK1016790A (GSK), capsaicin, pregnenolone sulfate (PS), lysophosphatidylcholine (LPC) and ionomycin calcium salt were purchased from Sigma-Aldrich; Allyl isothiocyanate (AITC) was purchased from KANTO; Camphor was purchased from Wako; Fura-2-acetoxymethyl ester was purchased from Invitrogen.

### Isolation and purification of mouse Schwann cells (SCs)

#### Isolation of mouse SCs

According to a method^48^ with some modifications, mouse SCs from postnatal day 1–3 (P1–3) mice were established. Sciatic nerves were isolated, cut into small pieces, and dissociated with 2.5 mg/mL dispase II (D4693, Sigma) and 0.5 mg/mL collagenase type IV (C5138, Sigma) for 30 min. The cells were resuspended in 10 mL basic growth medium containing Dulbecco’s modified essential medium (DMEM), 10% inactivated horse serum (HS; 26050070, Gibco), 4 mM L-glutamine (25030–081, Sigma), 1% penicillin–streptomycin (15140-122, Invitrogen), 2 ng/mL human heregulin-beta 1 (396-HB-050, R&D) and 0.5 μM forskolin (F6886, Sigma), plated in 100 mm Petri dishes pre-coated with 100 μg/mL poly-L-lysine (P1274, Sigma) and 10 ng/mL laminin (L2020, Sigma) and maintained at 37 °C in a 5% CO_2_ humidified incubator. Medium was refreshed every two days. According to a method^49^ with some modifications, mouse SCs from adult mice were established. Sciatic nerves were isolated from 15-week-old mice, cut into 10 mm segments and explanted into 30 mm dishes containing 750 μL high-glucose DMEM with 10% heat-inactivated FBS to facilitate myelin removal and cell recovery^50^. Medium was refreshed every two days. After 10 days, nerve explants were dissociated with 2.5 mg/mL dispase II and 0.5 mg/mL collagenase type IV for 30 min. Subsequent steps were identical to those described for postnatal SCs.

#### Purification of Schwann cells

After SC cultures had reached approximately 80% confluence, contaminating fibroblasts were removed by a complement reaction using Thy 1.1 antibodies^51^. Briefly, SCs were washed once with pre-warmed PBS then digested with 0.05% trypsin for 1–2 min. Cell suspensions were collected and washed twice with pre-warmed DMEM/10% HS by centrifugation. The cells were resuspended in 500 μL pre-cooled DMEM/10% HS containing 30 ng/μL anti-mouse Thy 1.1 antibody (MCAO2R, Bio-Rad) and incubated on ice for 1 h to allow antigen and antibody interaction. Antibody solution was removed by centrifugation and the resulting cell pellet was resuspended in 600 μL DMEM medium containing 200 μL rabbit serum complement (234400, Merck Millipore) and incubated at 37 °C for 1 hr. The cell pellet was collected and washed with pre-warmed DMEM by centrifugation. The cells were resuspended in SC growth medium (55) containing basic growth medium, 10 ng/mL human-basic fibroblast growth factor (GF003, Merck Millipore) and 20 μg/mL bovine pituitary extract (16500100, Cosmo Bio) before plating into a new dish pre-coated with 100 μg/mL poly-L-lysine and 10 ng/mL laminin. SC purity was determined to be >98% by S100 immunostaining. Purified SCs were used in subsequent experiments.

### RT-PCR

Total RNA was isolated from purified primary SCs using Sepasol-RNA I Super G (09379-84, Nacalai Tesque). Reverse transcription was performed using Super Script III reverse transcriptase (18080-085, Invitrogen) according to the manufacturer’s instructions. RNA concentration and quality were assessed using a Nanodrop (Isogen Life Science, Belgium)^52^. To investigate TRPV4 mRNA expression, cDNA fragments were amplified using EmeraldAmp MAX PCR Master Mix (DS-RR320A, TaKaRa) with PCR primers designed using Primer-BLAST from the National Center for Biotechnology Information (Table S1). All primers spanned an exon-exon junction. The PCR products were confirmed by electrophoresis on a 2% agarose gel containing ethidium bromide.

### Immunostaining

For immunostaining analysis of cultured SCs, cells were cultured on poly-L-lysine/laminin-coated glass coverslips. Cells were washed with PBS and fixed with pre-cooled 4% paraformaldehyde for 10 min and washed in PBS. Cells were incubated with blocking buffer (PBS containing 0.25% Triton X-100 and 1% bovine serum albumin) for 30 min at room temperature (RT), and then incubated overnight at 4 °C with primary antibody diluted in blocking buffer. After 3 washes with blocking buffer, secondary antibody (1:1,000, diluted in blocking buffer) was applied for 1 h at RT. Cells were then incubated in DAPI solution (D212, Wako) for another 10 min, followed by 3 washes with PBS. Images were obtained with a fluorescence microscope (BZ9000; Keyence, Osaka, Japan).

For immunostaining analysis of tissue sections, mice were euthanized by isoflurane and perfused with 4% pre-cooled paraformaldehyde. Distal sciatic nerves 1 mm away from the cut site were collected and post-fixed in 4% paraformaldehyde at 4 °C for 16 h. Fixed sciatic nerves were immersed in 30% sucrose at 4 °C for 24 h, embedded in optimal cutting temperature (OCT) compound, then sectioned to a thickness of 10 μm. Subsequent staining steps were identical to the abovementioned immunostaining analysis for cultured SCs. Images were obtained with a laser-scanning confocal microscope (LSM-510; Carl Zeiss).

The following primary antibodies were used: anti-S100 antibody (1:200; ab868, Abcam), anti-rabbit Myelin Protein Zero (1:200; ab31851, Abcam), anti-rat myelin basic protein (1:200; MAB386, Merck Millipore), anti-mouse MAG (1:300; sc-166848, Santa Cruz Biotechnology), anti-chicken GFAP (1:400; ab4674, Abcam), and anti-rabbit TRPV4 (1:500; CB-ACC-034, Alomone). The following secondary antibodies were used: goat anti-rat IgG Alexa Fluor^®^ 488 (a11006, Invitrogen), goat anti-chicken IgY (H + L) Alexa Fluor^®^ 647 (ab150171, Abcam), goat anti-chicken IgY (H + L) Alexa Fluor^®^ 488 (A11039, Invitrogen), donkey anti-rabbit IgG Alexa Fluor^®^ 488 (A21206, Invitrogen), and goat anti-mouse IgG (H + L) Alexa Fluor^®^ 546 (A11030, Invitrogen).

### Western blotting

Total protein was extracted from purified SCs or sciatic nerves that were washed with PBS 3 times and dissociated in lysis buffer containing 10 mM Tris-HCl, 150 mM NaCl, 1 mM EDTA-2Na, 1% Nonidet P-40, 1 mM Na_2_VO_4_, 10 mM NaF and Protease Inhibitor Cocktail (11873580001, Roche) for 30 min on ice. The supernatants were collected by centrifugation. Total protein was denatured and used for western blotting. The following primary antibodies were used in western blots: anti-rabbit Myelin Protein Zero (ab31851, Abcam), anti-rat myelin basic protein (MAB386, Merck Millipore), anti-mouse MAG (sc-166848, Santa Cruz Biotechnology), anti-rabbit TRPV4 (CB-ACC-034, Alomone), anti-rabbit EGR2 (ab108399, Abcam), anti-rabbit c-Jun mAb (9165, Cell Signaling Technology), and anti-mouse vinculin antibody (VIN-11-5, Sigma). The following secondary antibodies were used for western blotting: HRP-conjugated GAPDH rabbit mAb (8884, Cell Signaling Technology), HRP-linked anti-rabbit IgG (7074, Cell Signaling Technology), HRP-linked anti-mouse IgG (7076, Cell Signaling Technology), and HRP-linked anti-rat IgG (112-035-062, Jackson).

### Calcium imaging

Purified SCs were cultured on poly-L-lysine/laminin-coated glass coverslips. After loading with 5 μM Fura-2-acetoxymethyl ester (Fura-2) for 1 hr, SCs were mounted in an open chamber and superfused with bath solution. The standard bath solution contained 140 mM NaCl, 5 mM KCl, 2 mM MgCl_2_, 2 mM CaCl_2_, 10 mM HEPES, 10 mM glucose at pH 7.4 adjusted with NaOH. For Ca^2+^-free bath solution, 5 mM EGTA was added instead of 2 mM CaCl_2_. All chemicals were dissolved in the standard bath solution. For Ca^2+^-free experiments, chemicals were dissolved in Ca^2+^-free bath solution. All experiments were performed at room temperature unless otherwise stated. For thermal stimulation experiments, SCs were maintained at 33 °C in a 5% CO_2_ humidified incubator 24 h before Fura-2 loading. Thermal stimulation was performed by increasing the bath temperature using pre-warmed standard bath solution (~37 °C). The temperature was monitored using a thermocouple (TC-344; Warner Instruments, Hamden, CT, USA) placed into the bath. Cytosolic free Ca^2+^ concentrations were measured by dual-wavelength Fura-2 microfluorometry with excitation at 340/380 nm and emission at 510 nm. The ratio image was calculated and acquired using the IP-Lab image processing system (Scanalytics, Milwaukee, WI, USA).

### Patch-clamp recording from cultured SCs

Purified SCs were cultured on glass coverslips. Patch pipettes were made from borosilicate glass (type 8250, Garner Glass Company, United States) using a five-step protocol and a P-97 micropipette puller (Sutter Instrument, United States) with a tip resistance of 4–6 MΩ. Currents were recorded using an Axopatch 200B amplifier (Molecular Devices, United States), filtered at 5 kHz with a low-pass filter and digitized with Digidata 1440 A (Axon Instruments, United States). Data acquisition was achieved with pCLAMP 10 software (Axon Instruments, United States). The standard bath solution was the same as described for the calcium imaging. The cesium chloride pipette solution contained 140 mM CsCl, 5 mM EGTA, 10 mM HEPES, pH 7.40 with CsOH. The holding potential was −60 mV, and the ramp-pulse was from −100 to +100 mV for 300 ms. All data and graphs were statistically analyzed using Origin Pro8 (OriginLab, United States).

### Patch-clamp recording from SCs in whole-mount sciatic nerve bundles

#### Ex vivo sciatic nerve preparation

Mice were euthanized by isoflurane, and sciatic nerve bundles from WT mice that were uninjured or at 5 days post-injury were dissected under a dissection microscope. Sciatic nerve bundles were affixed in a recording chamber by a tissue anchor and submerged in a normal Krebs solution that contained 117 mM NaCl, 3.5 mM KCl, 1.2 mM MgCl_2_, 2.5 mM CaCl_2_, 1.2 mM NaH_2_PO_4_, 25 mM NaHCO_3_, and 11 mM glucose saturated with 95% O_2_ and CO_2_. The recording chamber was mounted on the stage of a microscope (BX51; Olympus) equipped with infrared-differential interference contrast (RI-DIC). The sciatic nerve bundles were exposed to 0.05% dispase II and collagenase in Krebs solution for 5 min and then washed with Krebs solution.

#### Whole-cell patch-clamp recording

Whole-cell patch-clamp recordings were performed on randomly-selected myelin sheaths of a SC from sciatic nerve bundles, which were prepared as shown above. The tip resistance was 4 to 6 MΩ after filling with internal solution containing 135 mM CsCl, 0.5 mM CaCl_2_, 2.4 mM MgCl_2_, 5 mM EGTA, 10 mM HEPES, 5 mM Na_2_ATP and 0.33 mM GTP-Tris salt; the pH was adjusted to 7.35 with CsOH. The whole-cell recording made from SCs in our whole-mount sciatic nerve preparation was normally stable for at least 2 h and no significant changes in basic electrophysiological parameters were observed. Signals in voltage-clamp experiments were amplified using an Axopatch 200B amplifier (Axon Instruments, United States), filtered at 2 kHz and sampled at 10 kHz using pCLAMP 10 software (Axon Instruments, United States). The holding potential was −60 mV, and the ramp-pulse was from −100 to +100 mV for 300 ms.

### Sciatic nerve cut injury

According to a method reported^53^, mice were first anesthetized with isoflurane, then the right sciatic nerve was exposed and cut at the sciatic notch (distal stump) before the skin was closed. Distal stumps that were 1 mm from the cut site were collected for analysis at various time points. Contralateral sciatic nerves were used as controls for immunohistochemistry and electron microscopy analysis. For western blot analysis, either contralateral or uninjured sciatic nerves were used as described in the text.

### Sciatic nerve cut injury in vitro

According to a method^7^ with minor modifications, sciatic nerves from 15-week-old WT and TRPV4KO mice were isolated, cut into 5 mm segments, and seeded in 35 mm plates. Nerve segments were cultured in DMEM medium containing 10% FBS and 1% penicillin–streptomycin in a 5% CO_2_ humidified incubator at 37 °C. After 7 days, the cultured nerve segments were collected and total protein was obtained for western blot analysis of TRPV4 protein expression.

### Walking track analysis

Mice were placed at the entrance of a corridor (50 cm × 5.5 cm) that contained a darkened cardboard box at the end. The floor of the corridor was covered with paper tape. The hind paws of mice were painted with ink, and mice walked straight through the corridor into the darkened box, leaving their footprints on the paper^54^. Three legible footprints were selected from the several footprints generated by each mouse, and the sciatic functional index (SFI) value was calculated^36^. Data on paw length (PL) and toe spread (TS) were collected from both the contralateral and ipsilateral hind legs. The value of SFI was calculated using the formula below:

$${\mathrm{Sciatic}}\,{\mathrm{functional}}\,{\mathrm{index}}\,\left( {{\mathrm{SFI}}} \right) = 118.91 \, * \, \frac{{{\mathrm{ETS}} - {\mathrm{NTS}}}}{{{\mathrm{NTS}}}} - 51.25 \, * \, \frac{{{\mathrm{EPL}} - {\mathrm{NPL}}}}{{{\mathrm{NPL}}}} - 7.57$$

ETS: toe spread (the distance between the first and fifth toe) of the ipsilateral hind paw; NTS: toe spread of the contralateral hind paw; EPL: paw length (the distance from the heel to the third toe) of the ipsilateral hind paw; NTS: paw length of the contralateral hind paw. In the presence of toe contracture, the print length was measured as the paw length plus the length from the proximal knuckle to the end of the toe^37^.

### Electron microscopy

According to a method described^55^, distal sciatic nerves tissue was preparation for imaging. The mice used for the walking track analysis were perfused with 2.5% glutaraldehyde and 4% paraformaldehyde in 0.1 M phosphate buffer. Distal sciatic nerves located 1 mm from the cut site were collected from WT and TRPV4KO mice, post-fixed for another 4 hr, and maintained at 4 °C overnight. Samples were treated with 2% OsO4 in 1.5% potassium ferricyanide for 1 h on ice, followed by sequential treatments with 1% thiocarbohydrazine for 20 min, 2% OsO4 for 30 min at RT, and 20 μM lead II aspartate solution containing 0.03 M L-aspartic acid solution (pH 5.2–5.5) for 30 min at 60 °C. After each of these treatments, the samples were washed 5 times with double-distilled water. The samples were then dehydrated with a graded series of ethanol and acetone and embedded in 7% carbon conductive resin over 3 days^56^. Some blocks were trimmed with an ultramicrotome and treated with gold sputtering to increase conductivity before imaging using field emission-SEM (Merlin or Sigma, Carl Zeiss AG) equipped with 3View (Gatan, Inc.). Other blocks were used to prepare ultrathin sections, which were imaged using transmission electron microscopy (H-7700, Hitachi High-Technologies). Image analysis was performed with Image J software. The g-ratio was calculated by dividing the axon diameter by the diameter of the axon including the myelin. The diameter was calculated by the measured perimeter divided by *π*.

### Statistics and reproducibility

Three independent experiments were performed. Data are presented as the mean ± SEM. Statistical analysis was performed with Origin Pro8 (RRID: SCR_014212; OriginLab, Haverhill, MA, USA). Significant changes were identified using a two-tailed *t-t*est, at 95% confidence interval, or one-way ANOVA followed by a Bonferroni post-hoc test with *p* < 0.05 considered as statistically significant (*p* values: *<0.05, **<0.01).

### Reporting summary

Further information on research design is available in the Nature Research Reporting Summary linked to this article.

## Supplementary information

Supplementary Information

Description of Additional Supplementary Files

Supplementary Data 1

Supplementary Data 2

Supplementary Movie 1

Supplementary Movie 2

## Data Availability

All data and materials used in the analysis are available in the main text with Figures and Supplementary Figures and Table. All uncropped gel images are available in the end of Supplementary Data 1. Statistical analysis data for the EM study are available in the Supplementary Data 2. Other data or information that supports the findings of this study are available from the corresponding author M.T (tominaga@nips.ac.jp) upon request.
