# Supplementary material for: Increased TRPV4 expression in non-myelinating Schwann cells is associated with demyelination after sciatic nerve injury
Source: Commun Biol. 2020 Nov 27;3:716. doi: 10.1038/s42003-020-01444-9 (PMC7695724; doi:10.1038/s42003-020-01444-9)
Supplement: Supplementary file 1 — Supplementary Information [file 42003_2020_1444_MOESM1_ESM.pdf]

Supplementary Figure 1. Expression of TRP channel genes in cultured mouse Schwann cells (SCs).

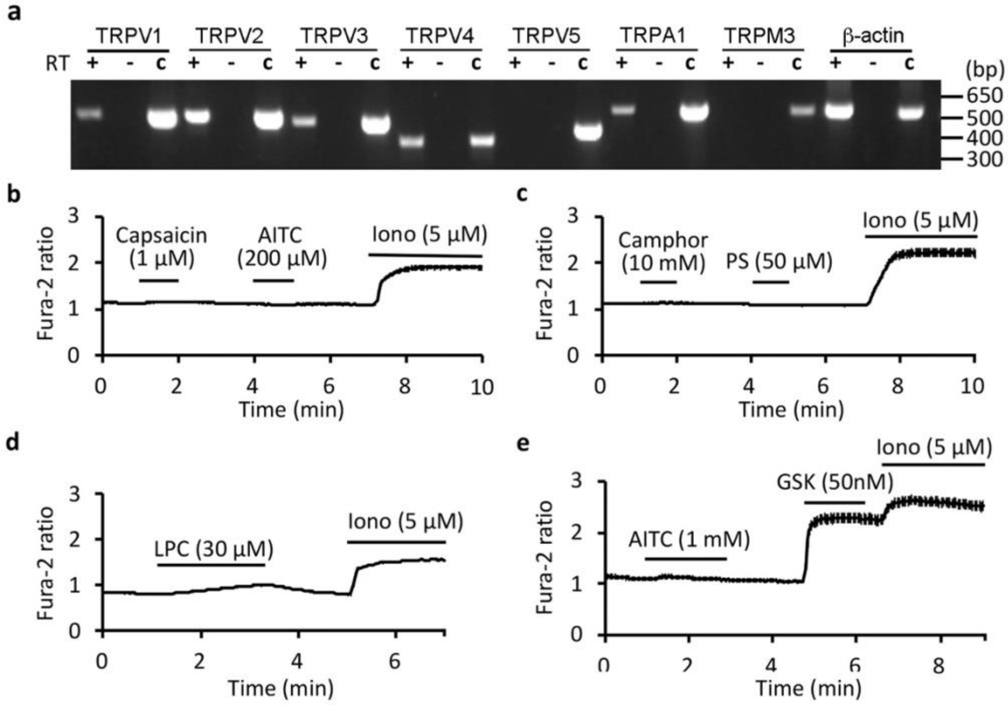

(a) RT-PCR for mRNA expression of TRPV1, TRPV2, TRPV3, TRPV4, TRPV5, TRPA1 and TRPM3 in purified primary Schwann cells (SCs) from P1-3 mice. RT (+) and (-) represent with and without reverse transcriptase treatment during cDNA sample preparation, respectively. "c" represents positive control with plasmid DNA as the DNA template for reverse transcription. β-actin mRNA was used as a loading control. The expected sizes of TRPV1, TRPV2, TRPV3, TRPV4, TRPV5, TRPA1, TRPM3 and β-actin are 548 bp, 552 bp, 523 bp, 404 bp, 464 bp, 590 bp, 577 bp, 556 bp and 573 bp, respectively.

(b-e) Mean Fura-2 ratios corresponding to intracellular Ca^{2+} concentrations in primary SCs isolated from P1-3 mice. Capsaicin, allyl isothiocyanate (AITC), camphor, pregnenolone sulfate (PS) and lysophosphatidylcholine (LPC) are agonists of TRPV1, TRPA1, TRPV3, TRPM3 and TRPV2, respectively. SCs were stimulated with capsaicin, AITC (b, n=30 cells), camphor, PS (c, n=27 cells), LPC (d, n=114 cells), AITC and GSK (e, n=25 cells) with the indicated concentrations in the presence of 2 mM extracellular Ca^{2+}. Data are presented as the mean ± SEM.

Supplementary Figure 2. WT and TRPV4KO mice show similar expression levels of P0 and MAG proteins at different ages.

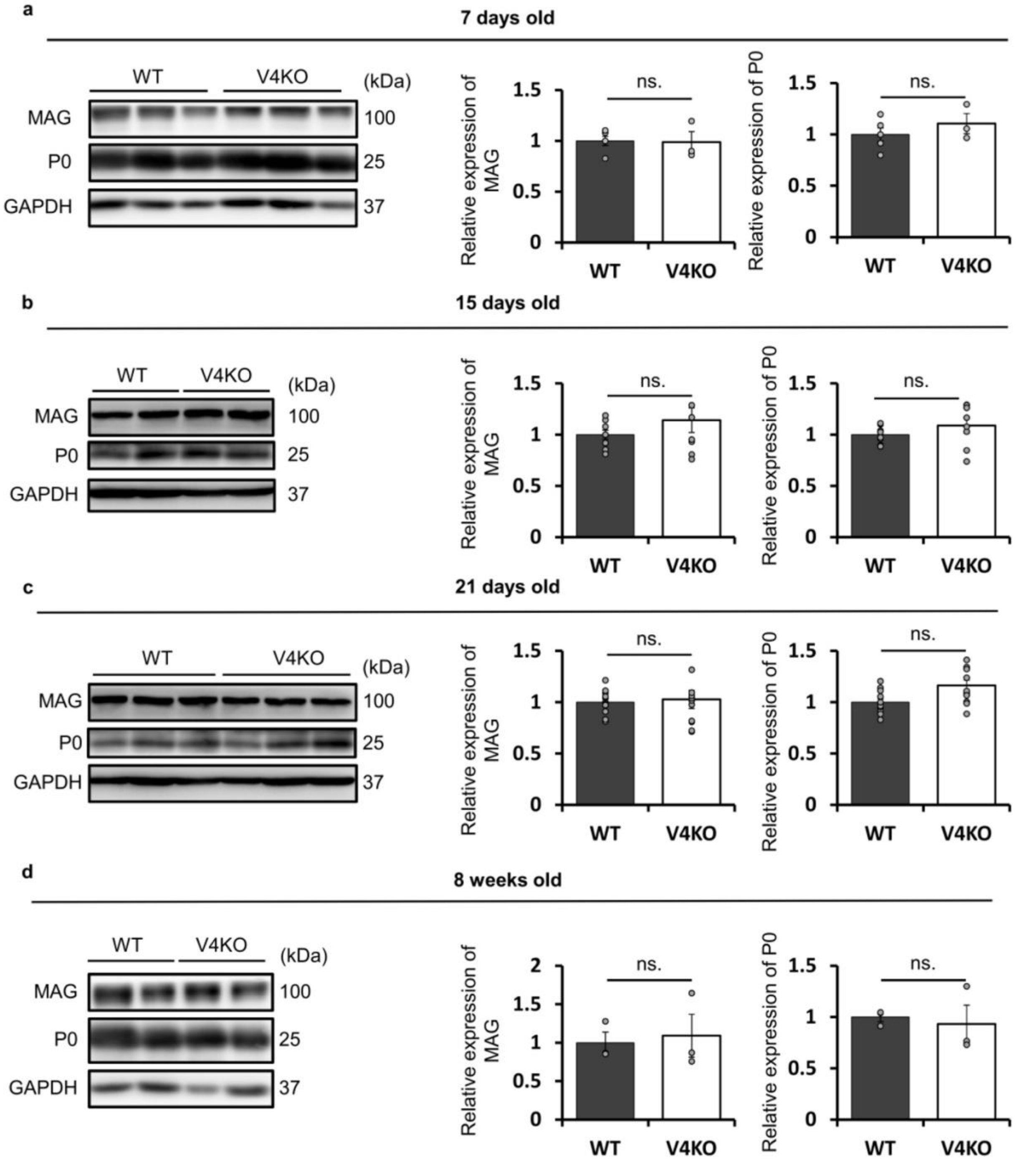

Representative western blot images of MAG and P0 in sciatic nerves from postnatal day 7 (a, left), postnatal day 15 (b, left) postnatal day 21 (c, left) and 8-week-old mice (d, left) from WT and TRPV4KO (V4KO) littermates. Comparison of expression levels normalized to GAPDH are shown at right. Data are presented as the mean ± SEM (5 WT and 3 V4KO mice were used for P7 mice; 6 WT and 6 V4KO mice were used for P15 mice; 9 WT and 8 V4KO mice were used for P21 mice; 3 WT and 3 V4KO mice were used for 8-week-old mice). A two-tailed *t*-test was used for comparison. ns. = not significant.

Supplementary Figure 3. NF 160, MAG and P0 protein expressions in WT sciatic nerves between 5 days and 35 days after injury.

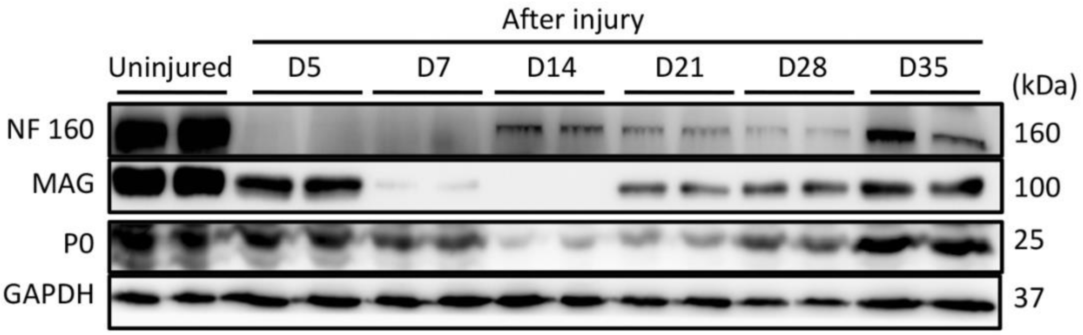

Representative western blot analysis of NF 160, MAG and P0 proteins in distal sciatic nerves from 15-week-old WT mice between 5 days (D5) and 35 days (D35) after injury. GAPDH was used as a loading control. n=2 mice, two independent experiments were performed.

Supplementary Figure 4. TRPV4 is detected in teased sciatic nerves from WT, but not TRPV4KO mice.

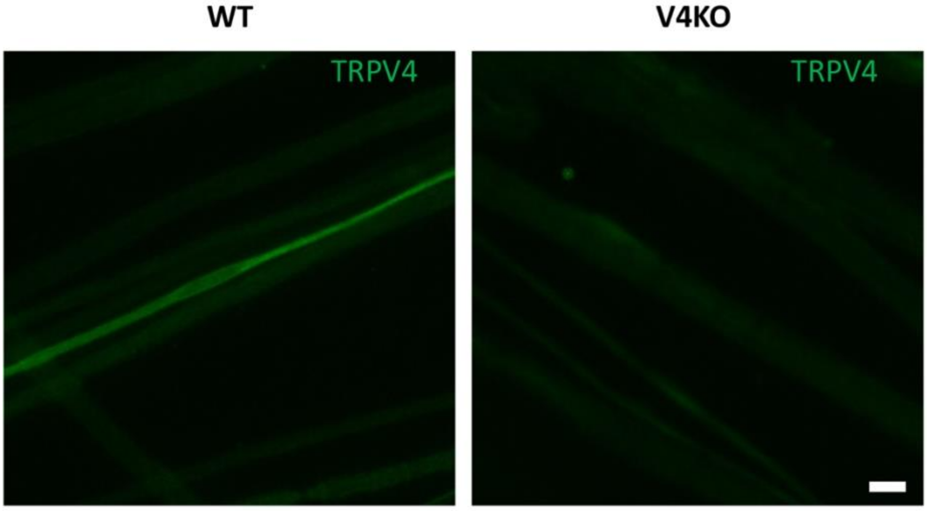

Immunostaining images of TRPV4 (green) in nerve fibers teased apart from the sciatic nerve of 15-week-old WT (left) and TRPV4KO (right) mice. Scale bar: 10 μm.

Supplementary Figure 5. Thinned myelin sheaths in TRPV4KO mice is independent of axon size.

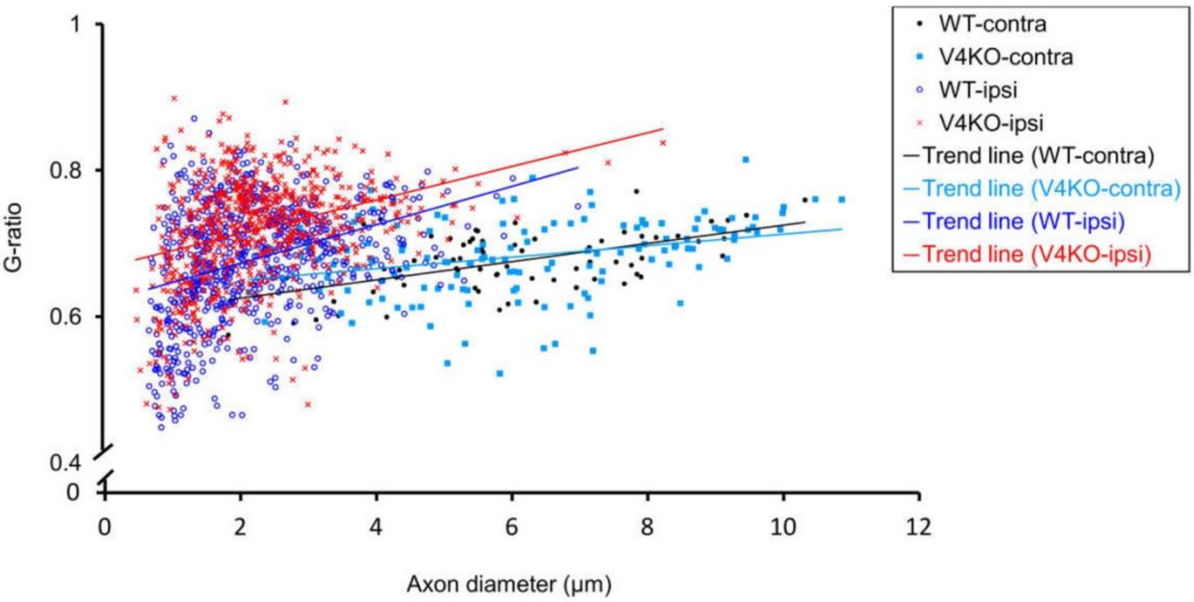

Scatter plot of G-ratios corresponding to individually measured axons in contralateral (n = 70 axons for WT, and n = 140 for TRPV4KO mice) and ipsilateral (n = 707 axons for WT, and n = 728 for TRPV4KO mice) distal sciatic nerves from WT (n=3) and TRPV4KO mice (n=3) 2 months after injury.

Supplementary Table 1.
Primers used for reverse transcription-PCR (RT-PCR)

Gene	Forward primer	Reverse primer
Trpv1	AACTCCACCCCACACTGAAG	TCGCCTCTGCAGGAAATACT
Trpv2	ACCGCATGGTGGTTTTAGAG	CTACAGCAAAGCCGAAAAGG
Trpv3	CATCACCCTGACCCTTGTCT	GCTGAAGCTGCCATAGGAAC
Trpv4	ACAACACCCGAGAGAACACC	CCCAAACTTACGCCACTTGT
Trpv5	GAGTTGGTGCCTCTCGCTAC	GGCAAAGGTGGCATAGGTAA
Trpv6	TCATTGAGCATGGAGCTGAC	GGTCACATAGGCCTCCTGAA
Trpa1	AACTCCTCAACCACCCTGTG	CTGAGGCCAAAAGCCAGTAG
Trpm3	GCCATTCTCTTTCCCAATGA	ACGAATTGAAGCGATCATCC
Actb	TGTTACCAACTGGGACGACA	AAGGAAGGCTGGAAAAGAGC

All primers span an exon-exon junction.
