# Supplementary material for: Increased TRPV4 expression in non-myelinating Schwann cells is associated with demyelination after sciatic nerve injury
Source: Commun Biol. 2020 Nov 27;3:716. doi: 10.1038/s42003-020-01444-9 (PMC7695724; doi:10.1038/s42003-020-01444-9)
Supplement: Supplementary file 2 — Description of Additional Supplementary Files [file 42003_2020_1444_MOESM2_ESM.pdf]

Description of Additional Supplementary Files

File Name: Supplementary Data 1

Description: The source data WB uncropped images

File Name: Supplementary Data 2

Description: Statistical analysis data for the EM study

File Name: Supplementary Movie 1

Description: Wild type mouse walking in the open field area

File Name: Supplementary Movie 2

Description: TRPV4KO mouse walking in the open field area
