# Supplementary material for: Increased TRPV4 expression in non-myelinating Schwann cells is associated with demyelination after sciatic nerve injury
Source: Commun Biol. 2020 Nov 27;3:716. doi: 10.1038/s42003-020-01444-9 (PMC7695724; doi:10.1038/s42003-020-01444-9)

**Fig 1c**

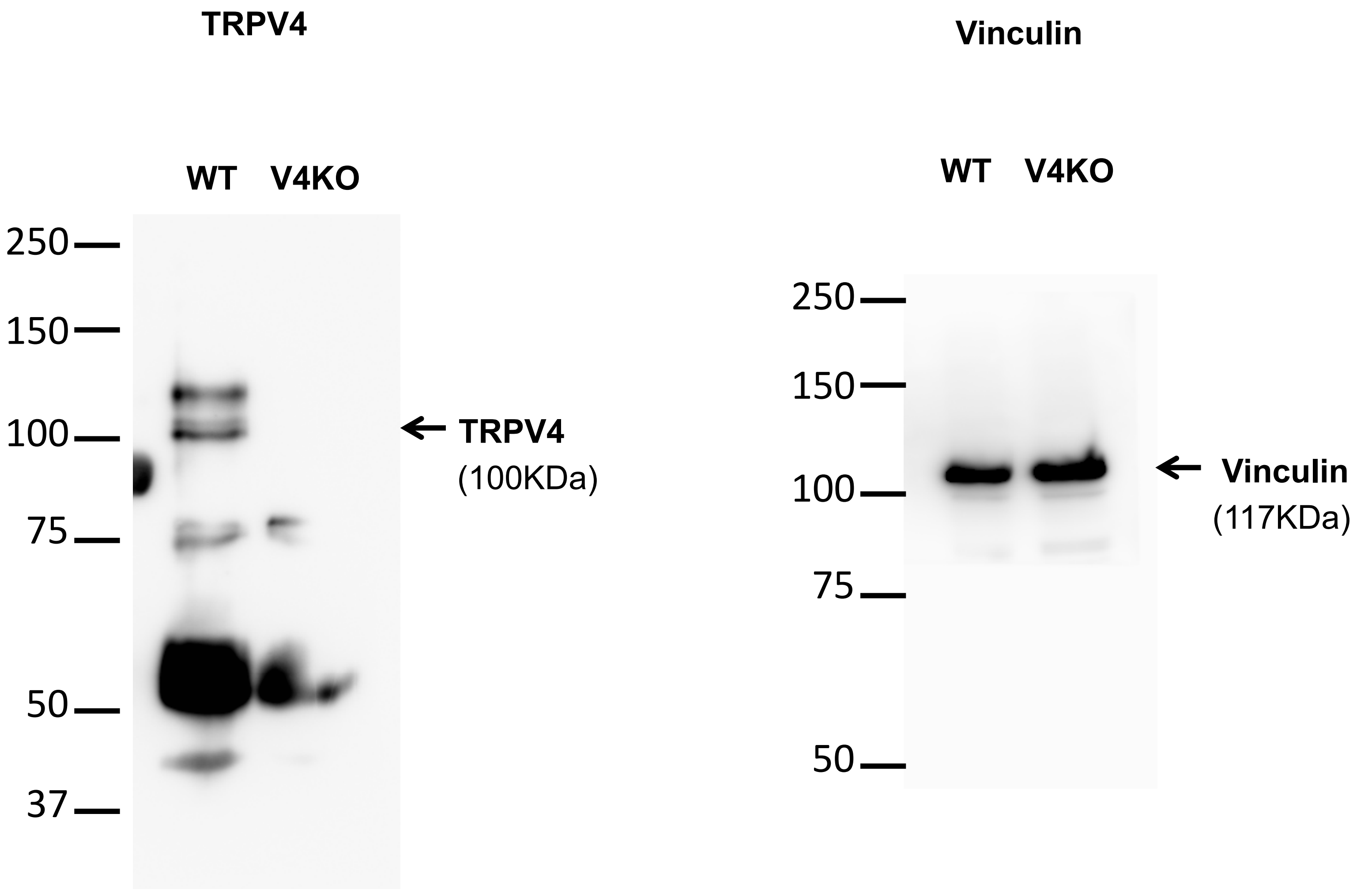
TRPV4
WT
V4KO
250
150
100
75
50
37
TRPV4
(100KDa)
Vinculin
WT
V4KO
250
150
100
75
50
Vinculin
(117KDa)

**Fig 1b**

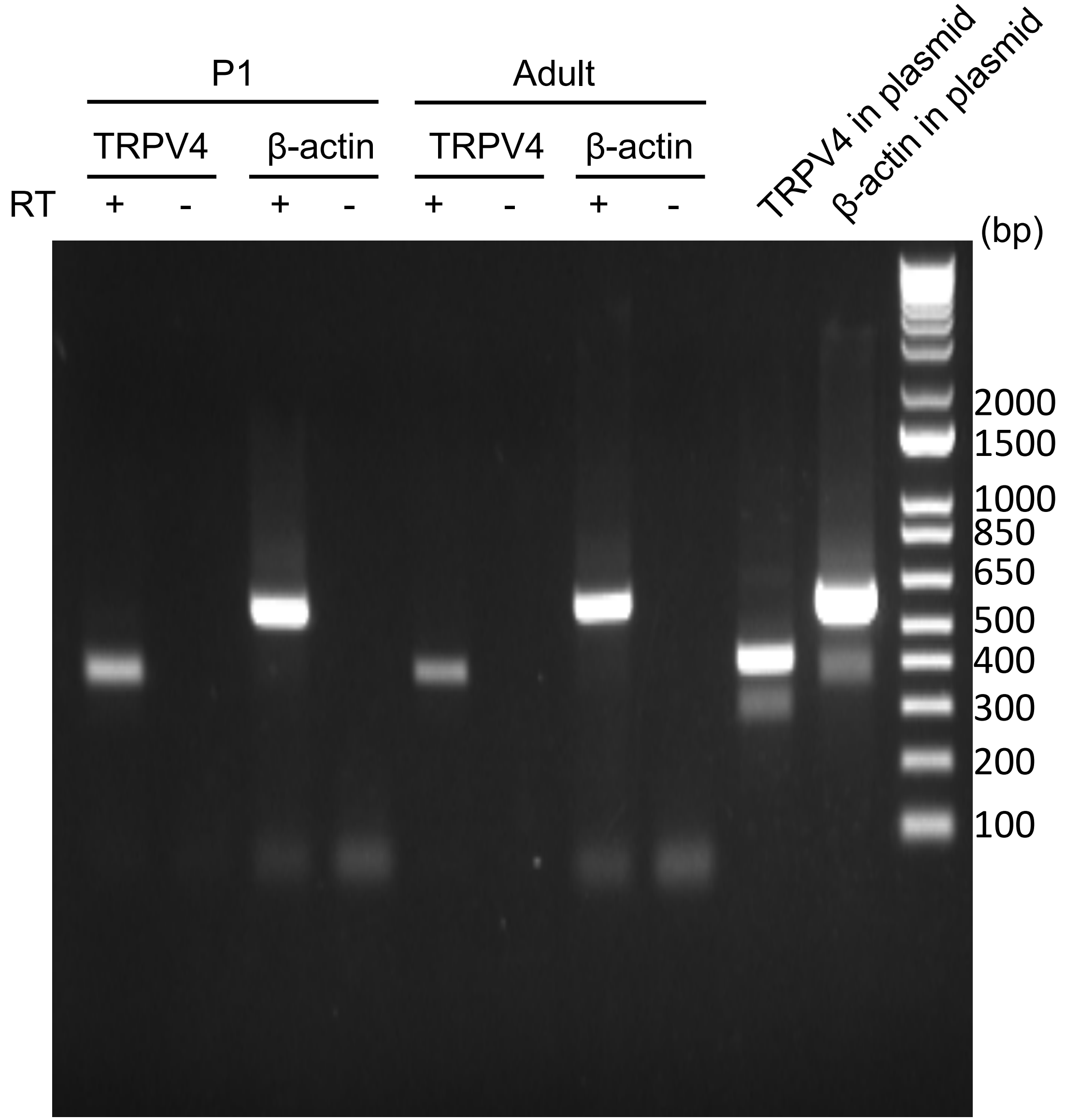

P1
Adult
TRPV4
β-actin
TRPV4
β-actin
RT
+
-
+
-
+
-
+
-
TRPV4 in plasmid
β-actin in plasmid
(bp)
2000
1500
1000
850
650
500
400
300
200
100

**Fig 3a**

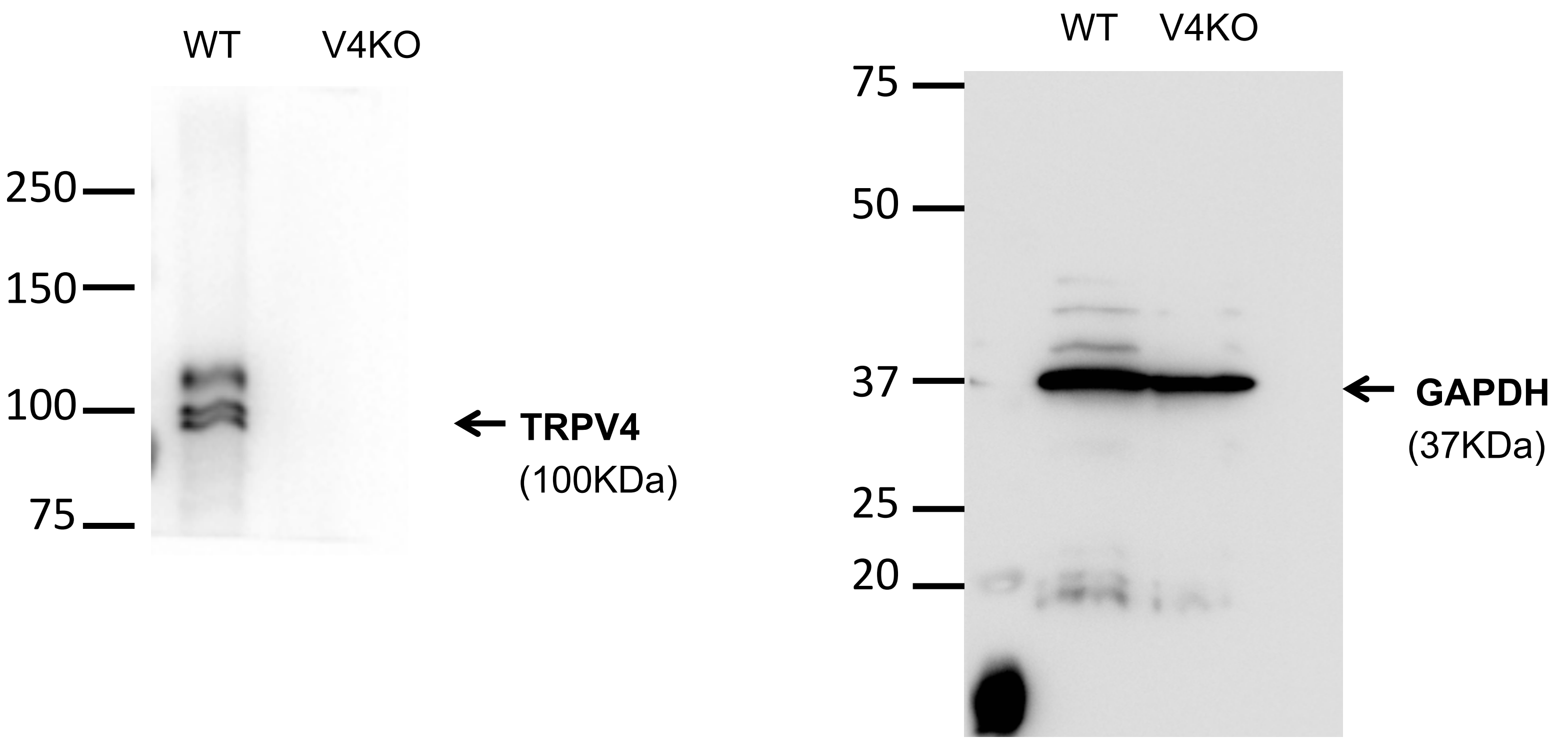
WT
V4KO
250
150
100
75
TRPV4
(100KDa)
WT
V4KO
75
50
37
25
20
GAPDH
(37KDa)

**Fig 3b**

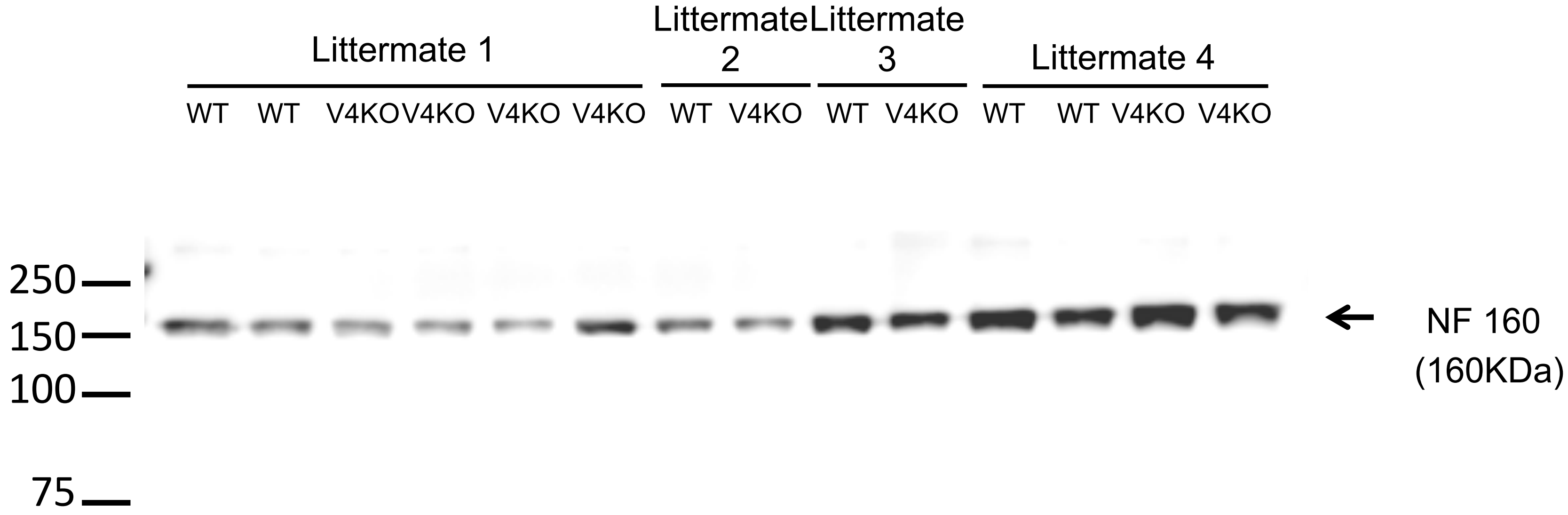
Littermate 1
Littermate 2
Littermate 3
Littermate 4
WT WT V4KO V4KO V4KO V4KO WT V4KO WT V4KO WT WT V4KO V4KO
250
150
100
75
NF 160
(160KDa)

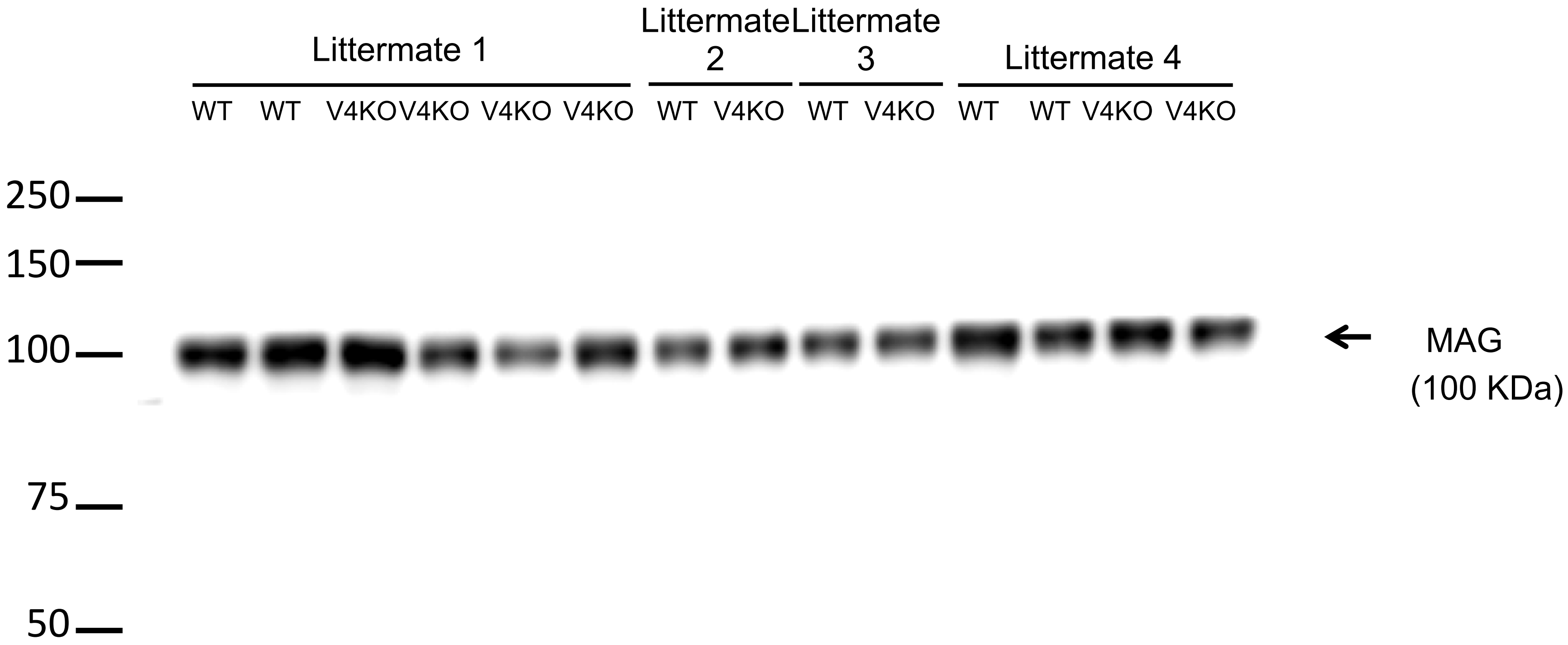
Littermate 1
Littermate 2
Littermate 3
Littermate 4
WT WT V4KO V4KO V4KO V4KO WT V4KO WT V4KO WT WT V4KO V4KO
250
150
100
75
50
MAG
(100 KDa)

**Fig 3b**

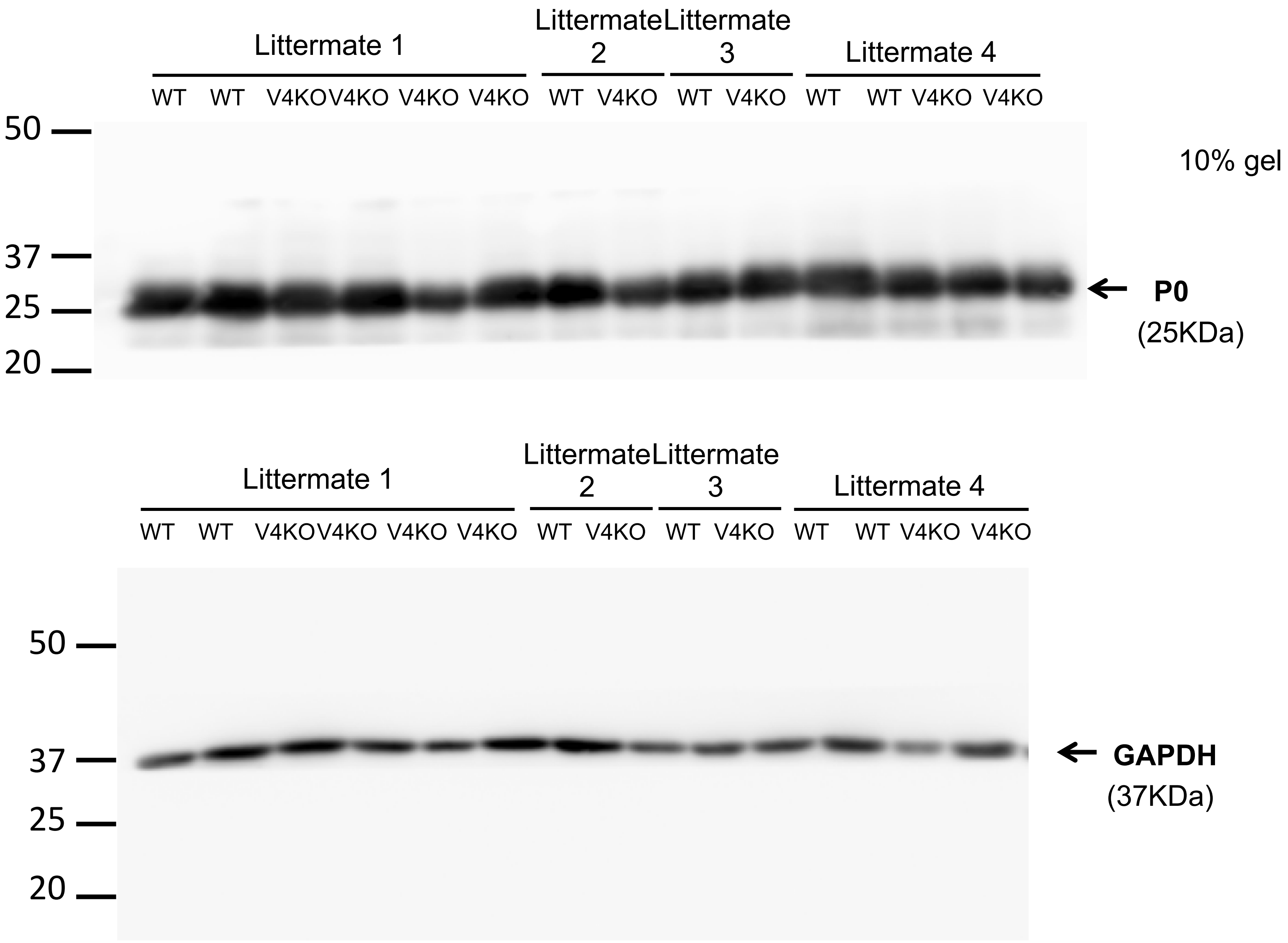
Littermate 1
Littermate 2
Littermate 3
Littermate 4
WT WT V4KO V4KO V4KO V4KO WT V4KO WT V4KO WT WT V4KO V4KO
50
37
25
20
10% gel
P0
(25KDa)
Littermate 1
Littermate 2
Littermate 3
Littermate 4
WT WT V4KO V4KO V4KO V4KO WT V4KO WT V4KO WT WT V4KO V4KO
50
37
25
20
GAPDH
(37KDa)

**Fig4 b - TRPV4 in vivo**

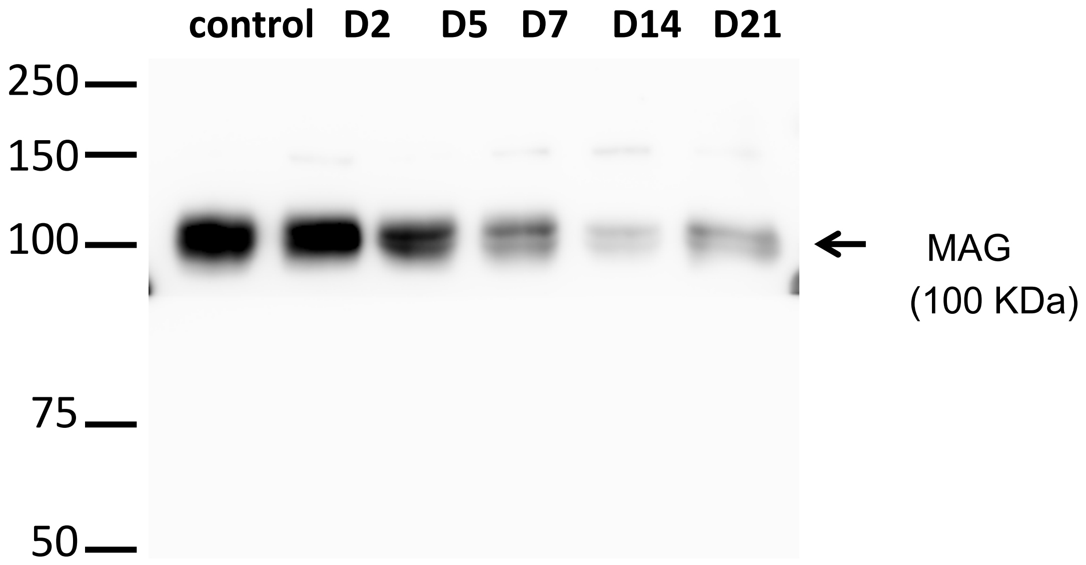


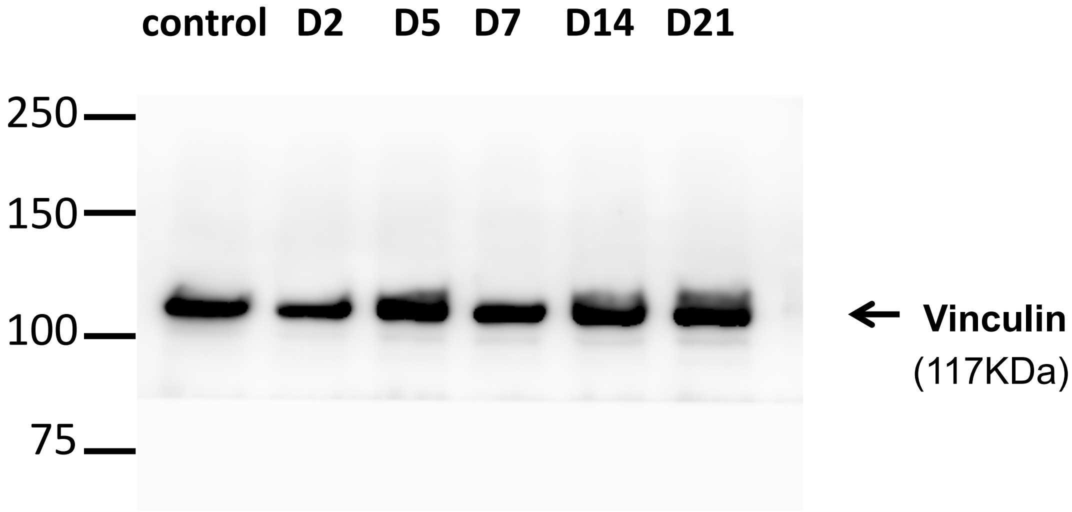

# Fig4 b and d- TRPV4 in vivo

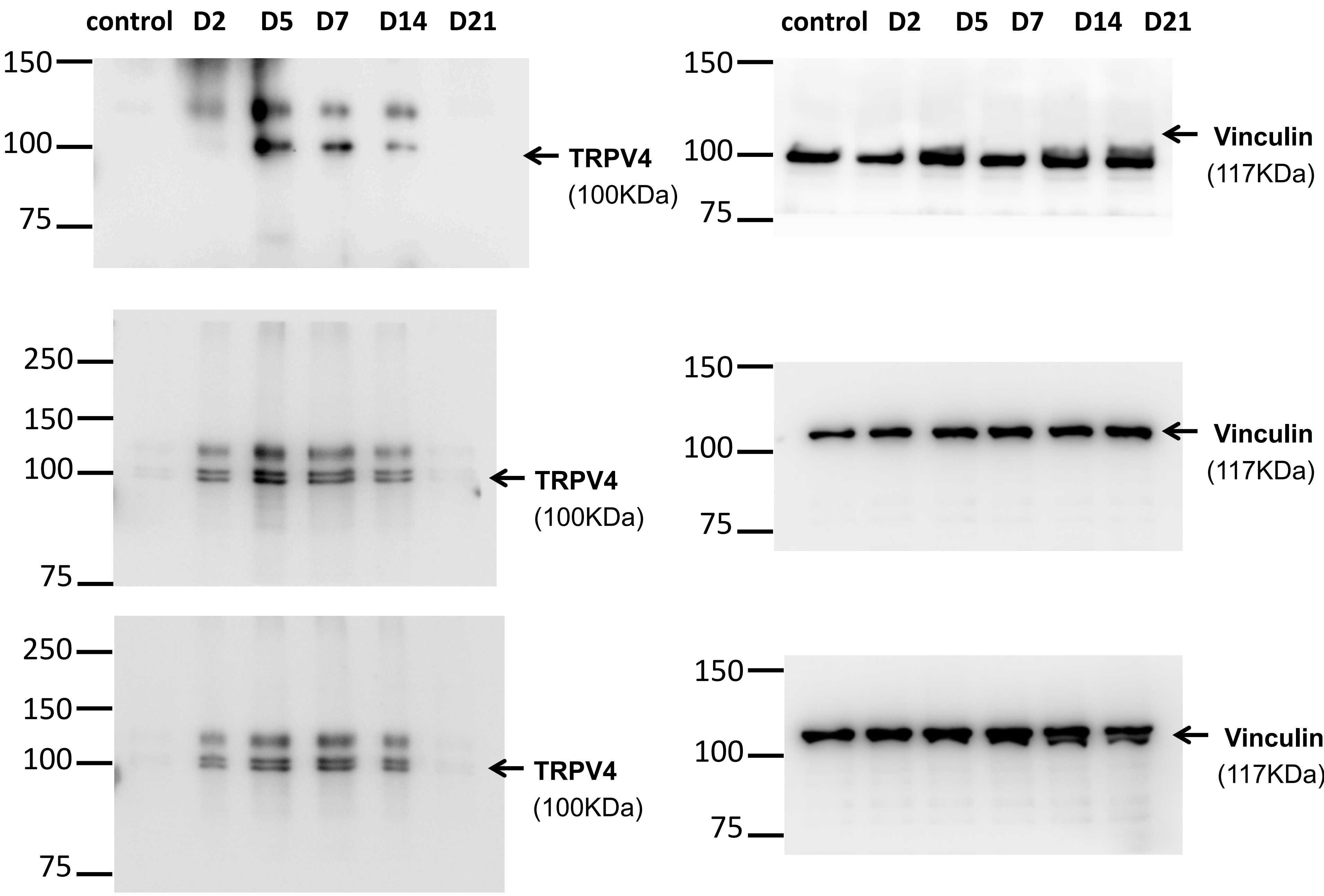

## Fig4 c krxo20, cJun, MAG in vitro

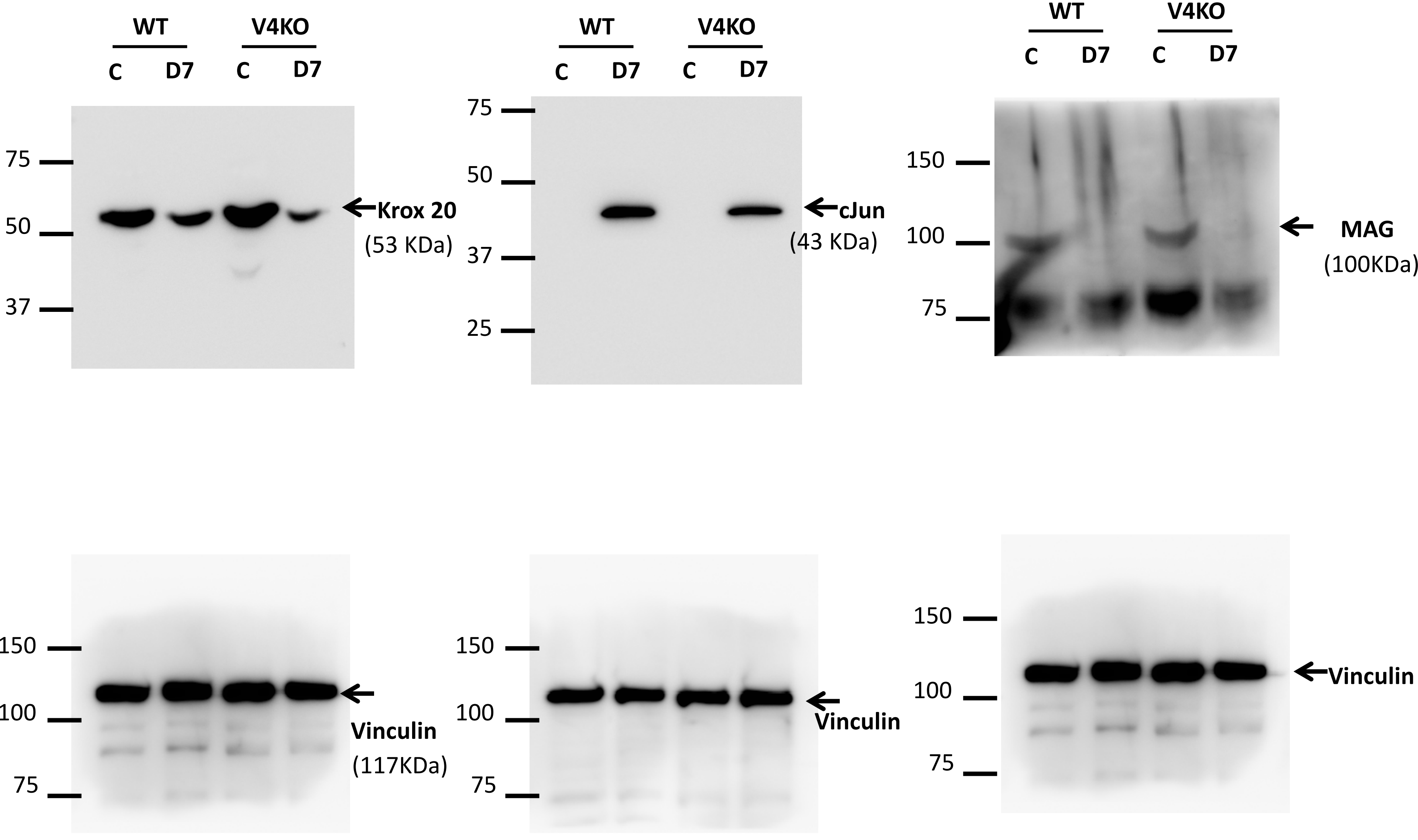

# Fig4 c and e- TRPV4 in vitro

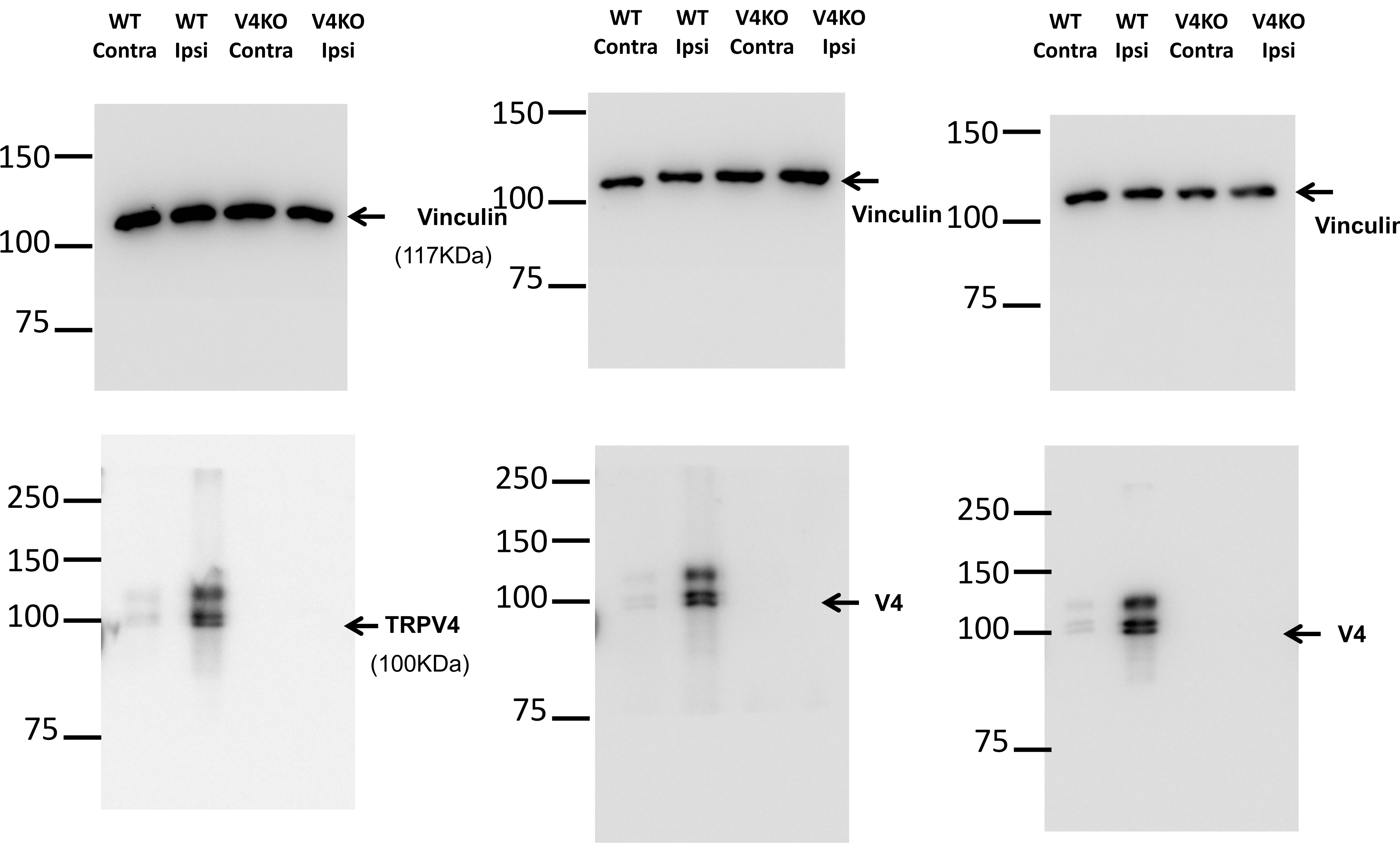

Fig6 a and b- cut D7

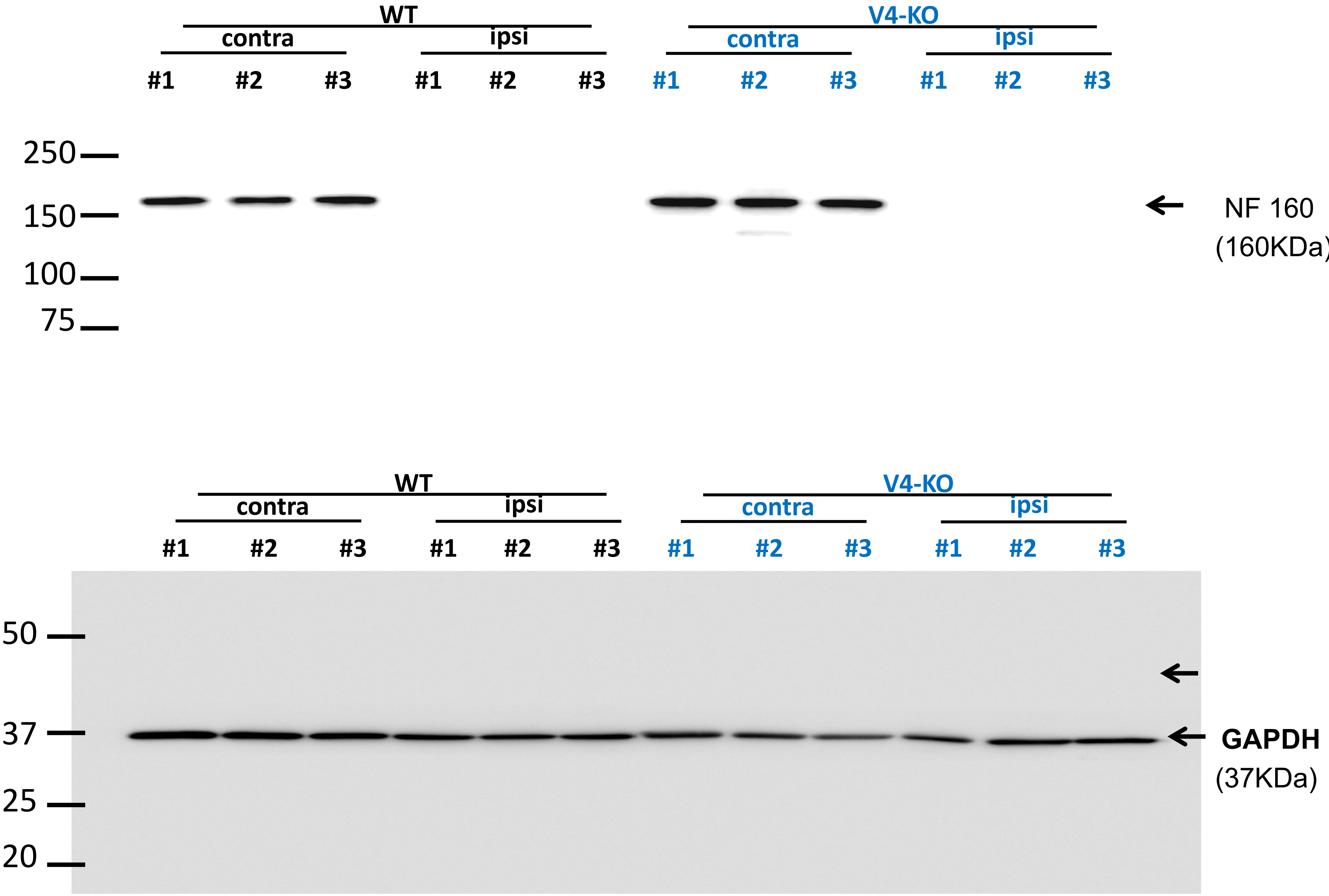

Fig6 a and b-  cut D7

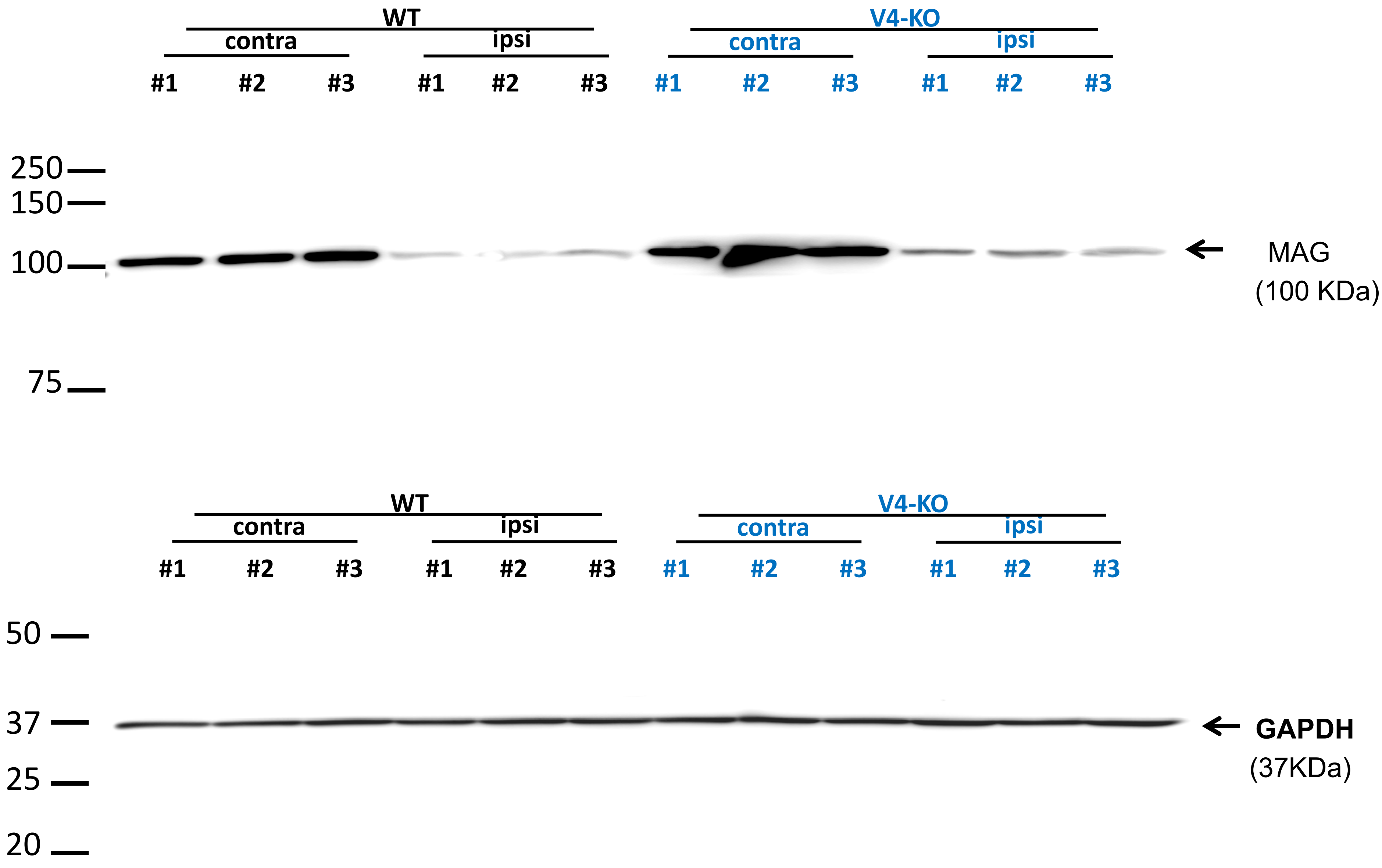

**Fig6 a and b- cut D7**

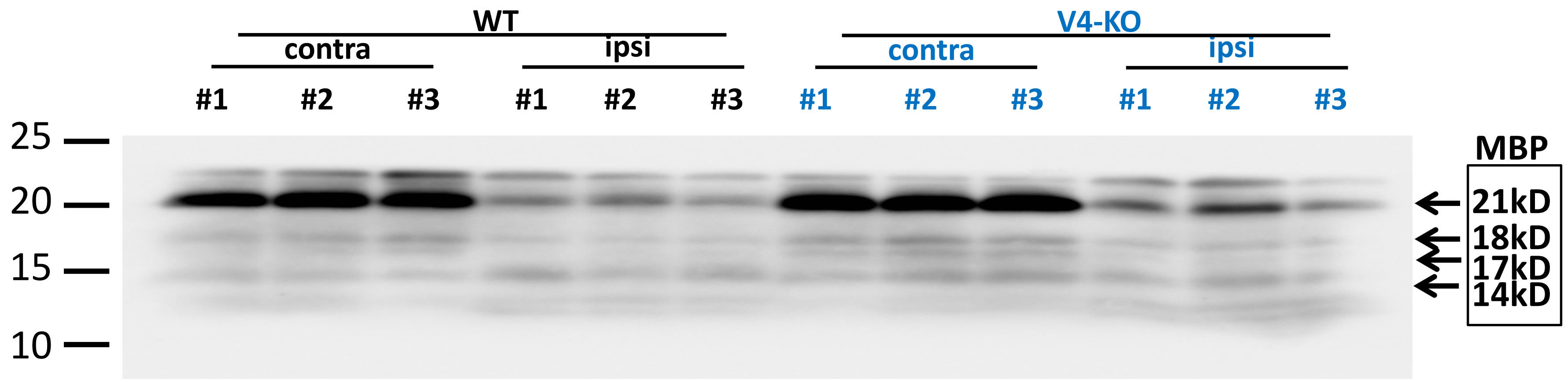


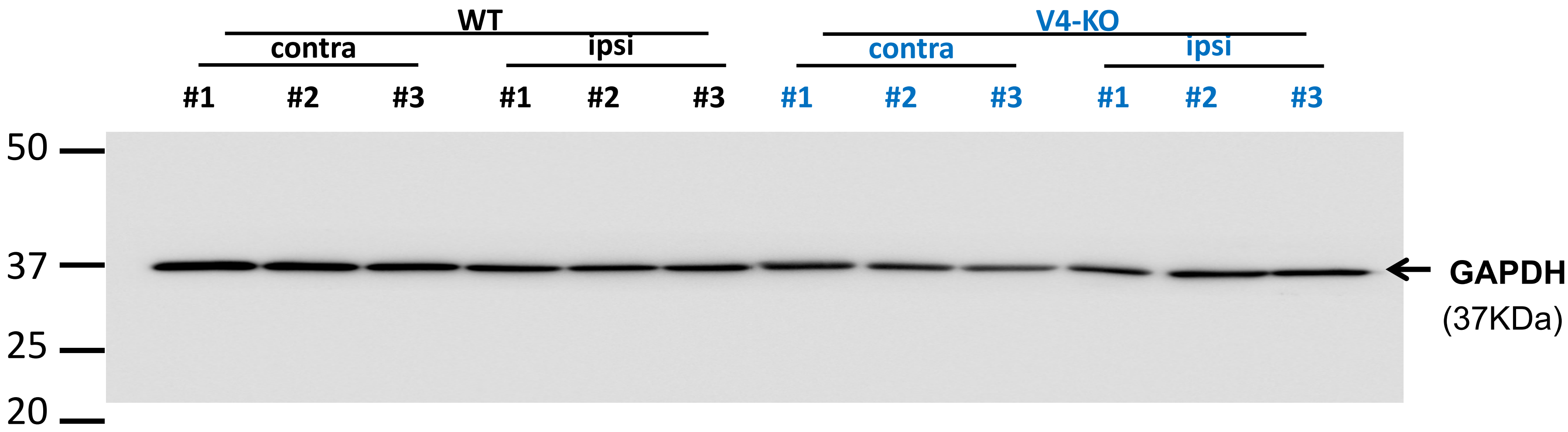

Fig6 a and b- cut D7

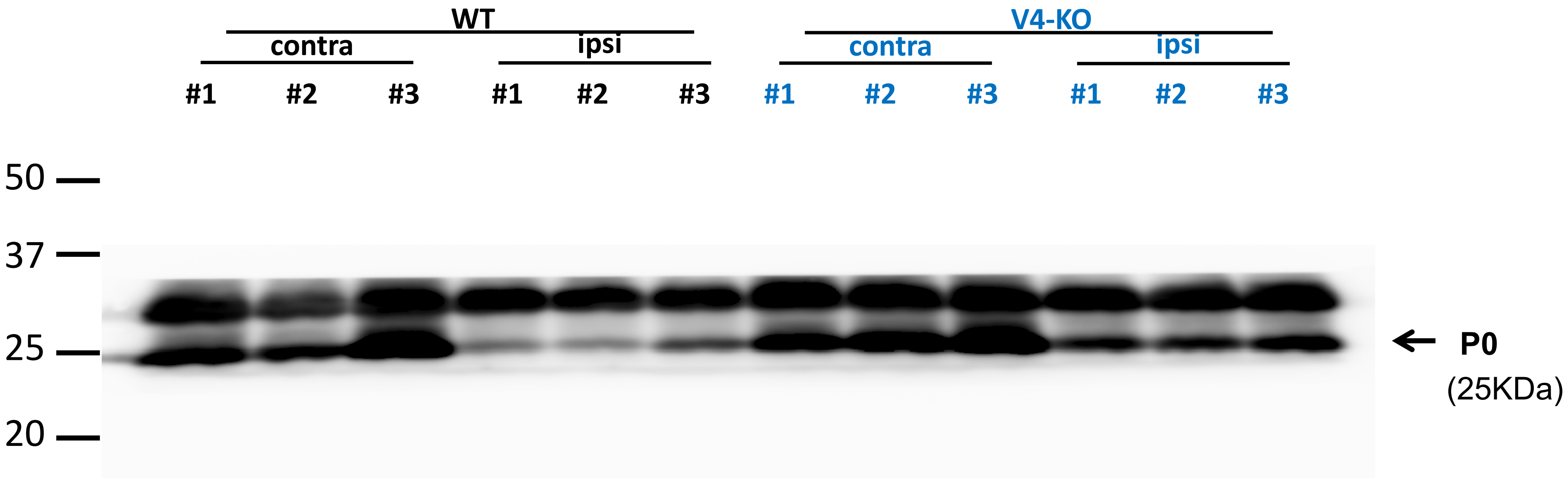


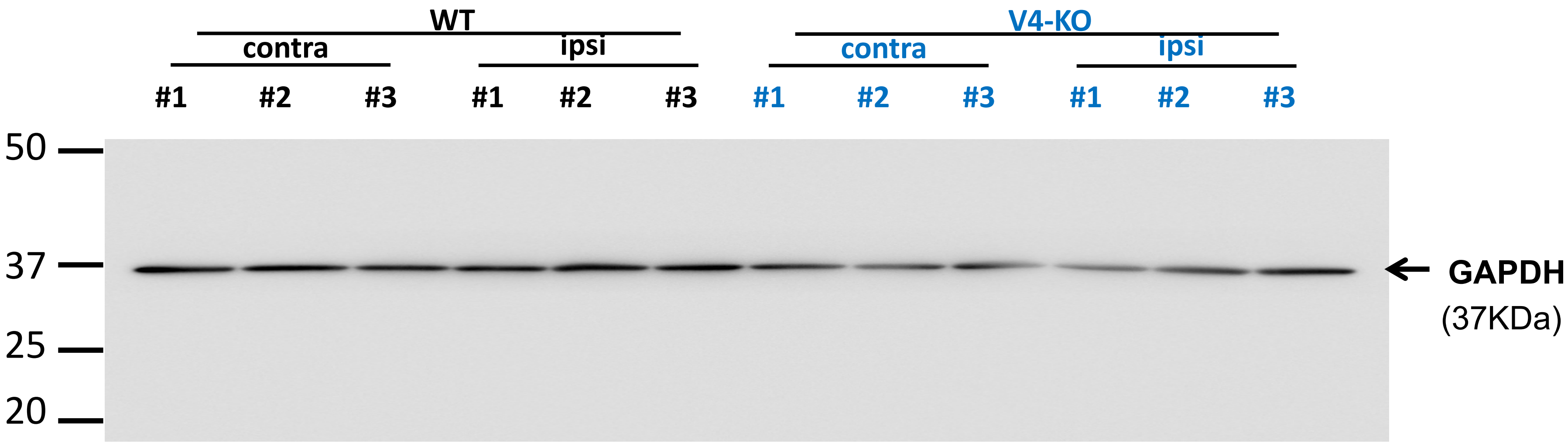

**Fig6 c and d- cut D7-M2**

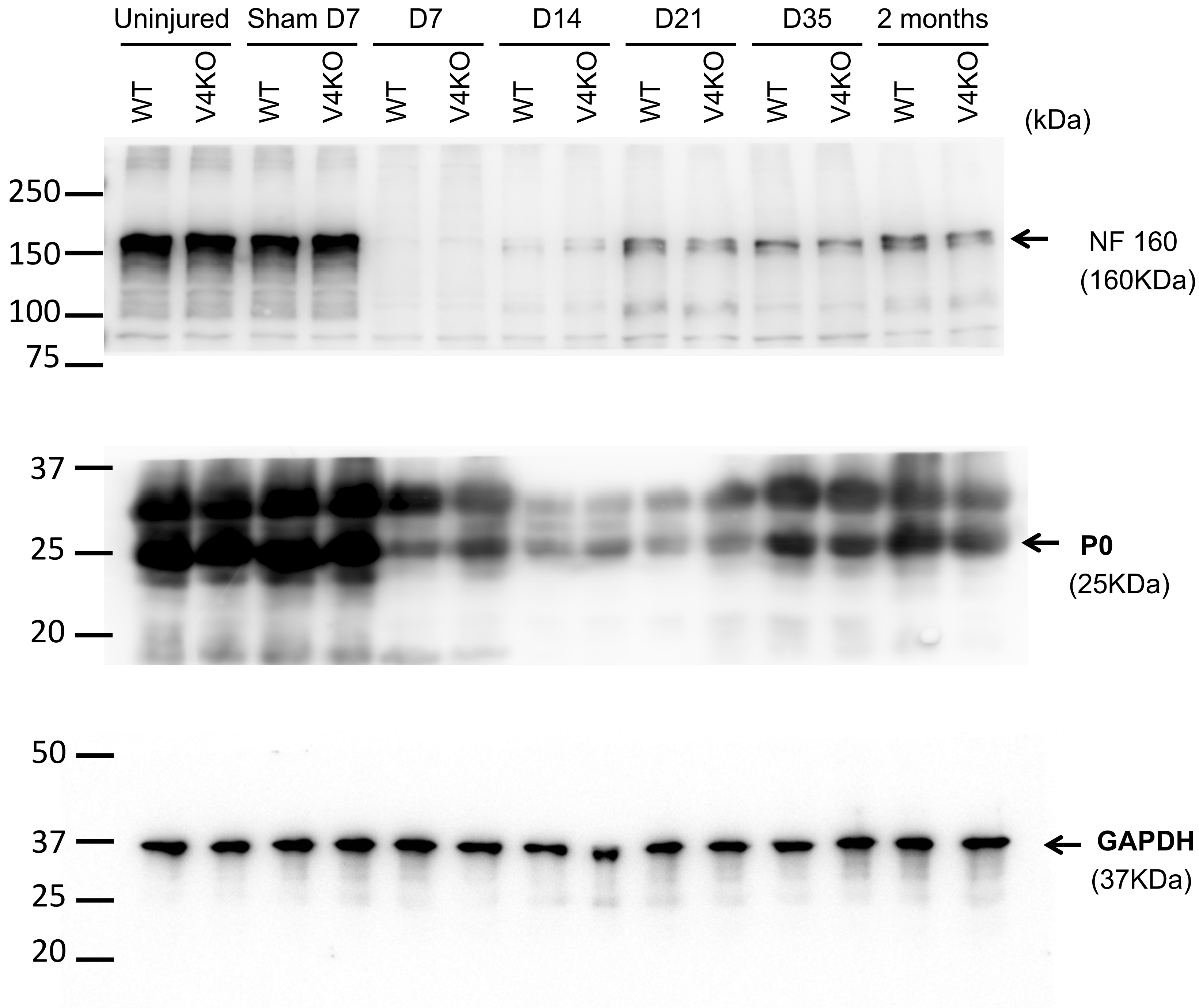

**Supplementary Fig 1**

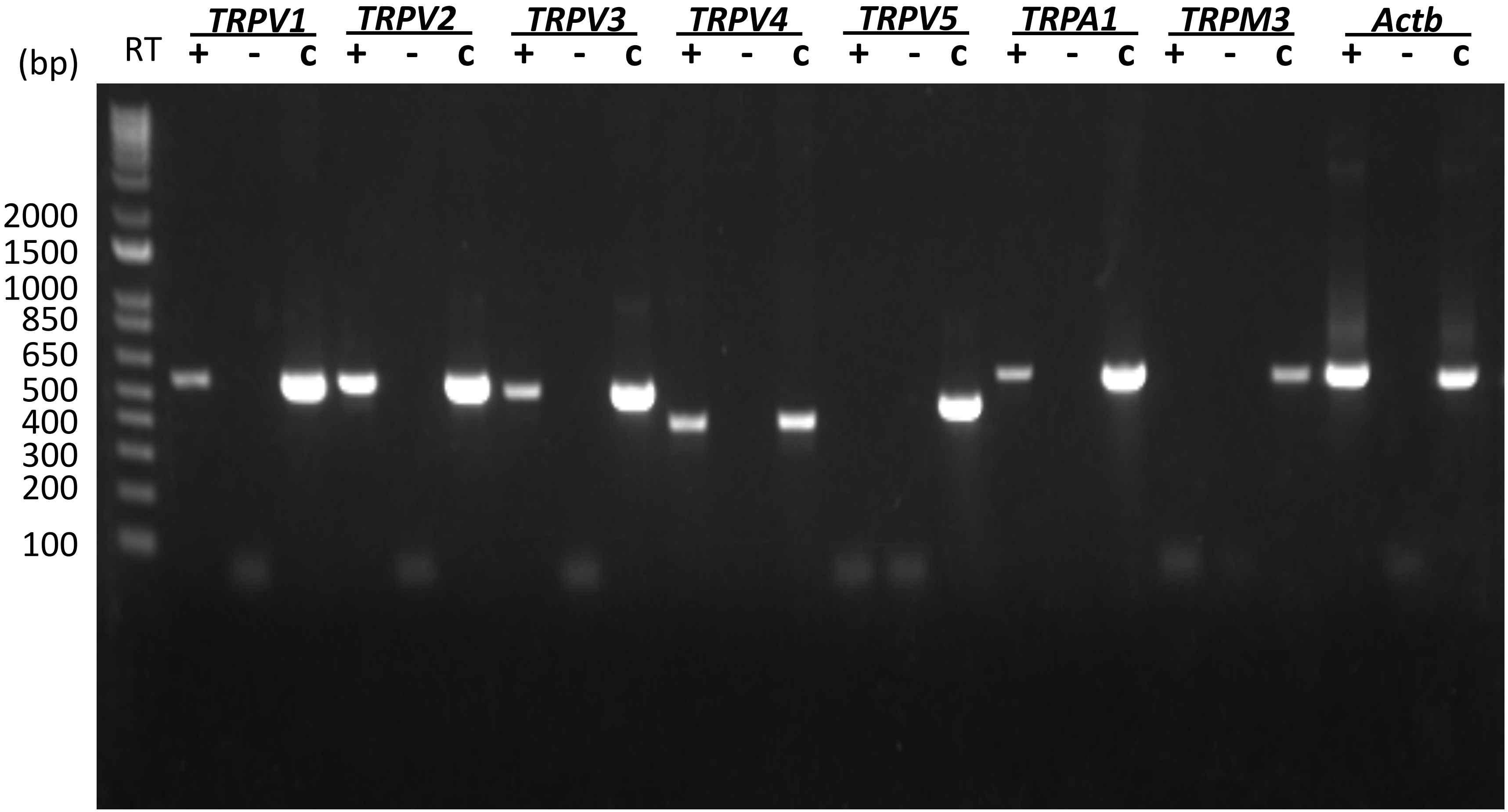

## Supplementary Fig 3

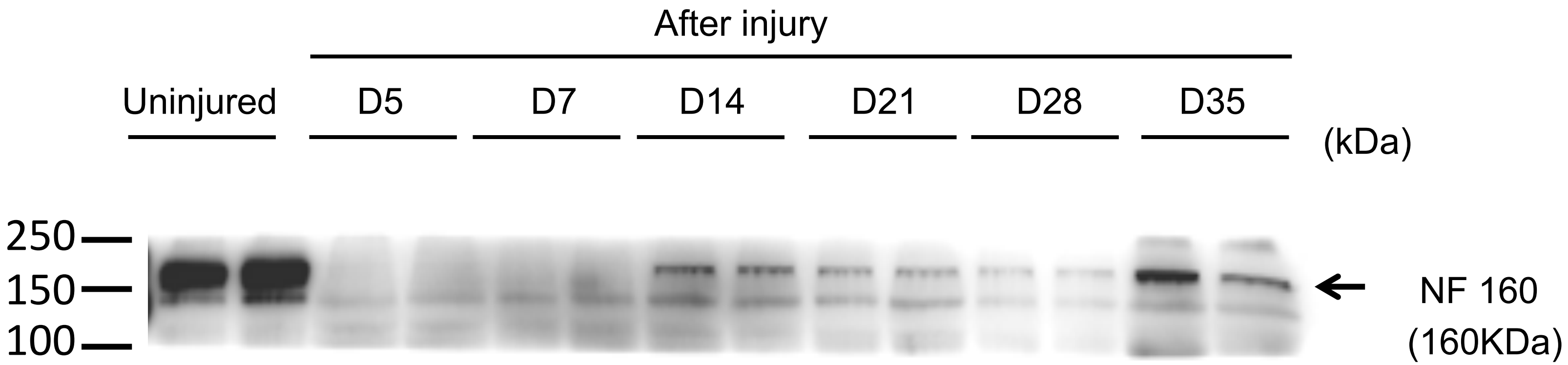


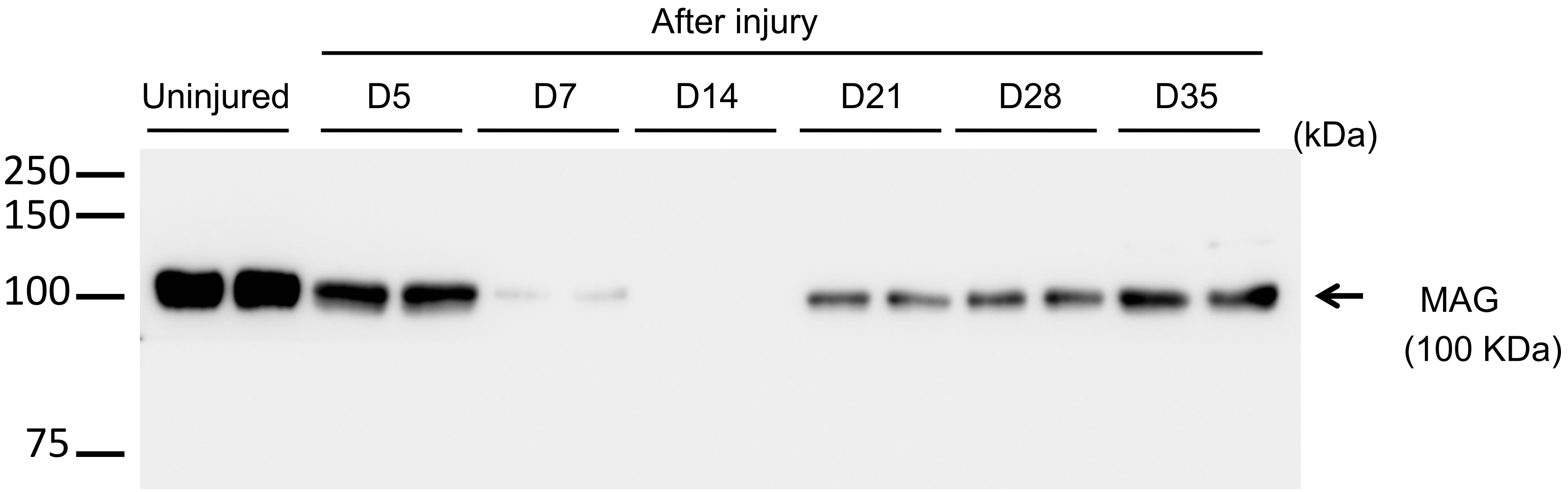

## Supplementary Fig 3

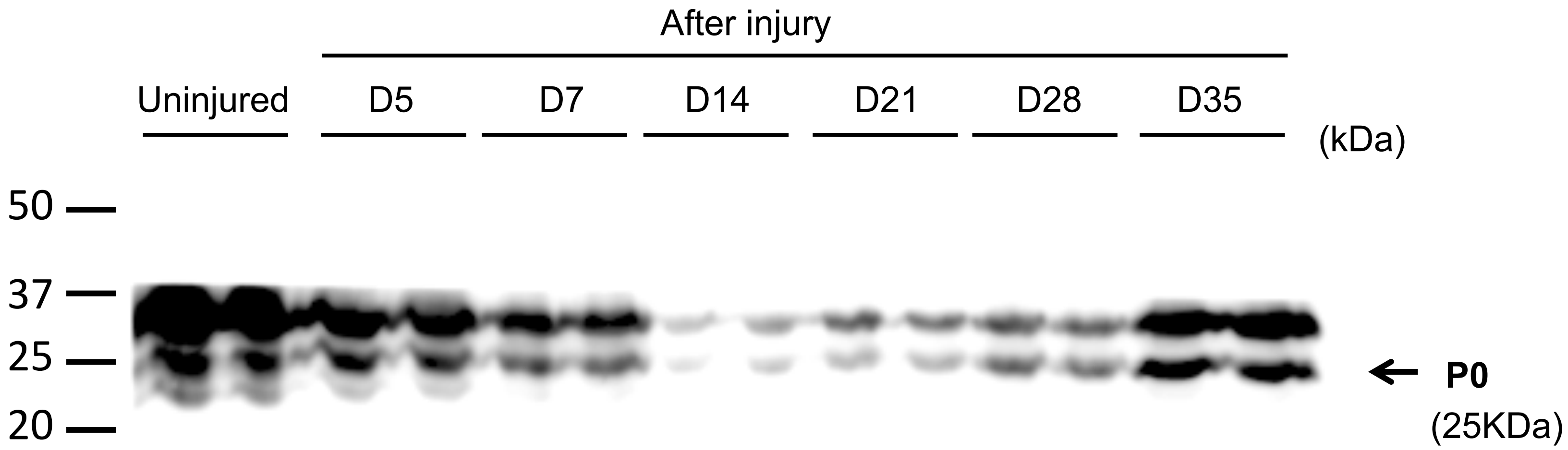


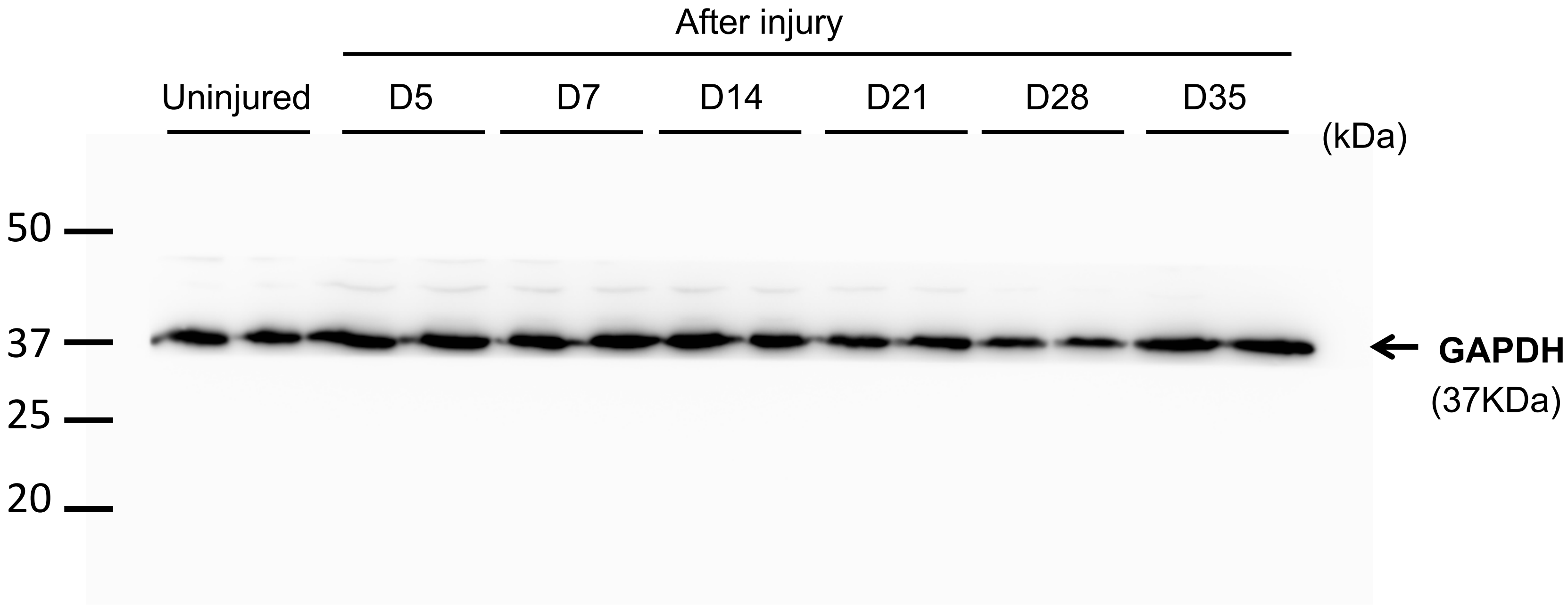

**Supplementary Fig 2**

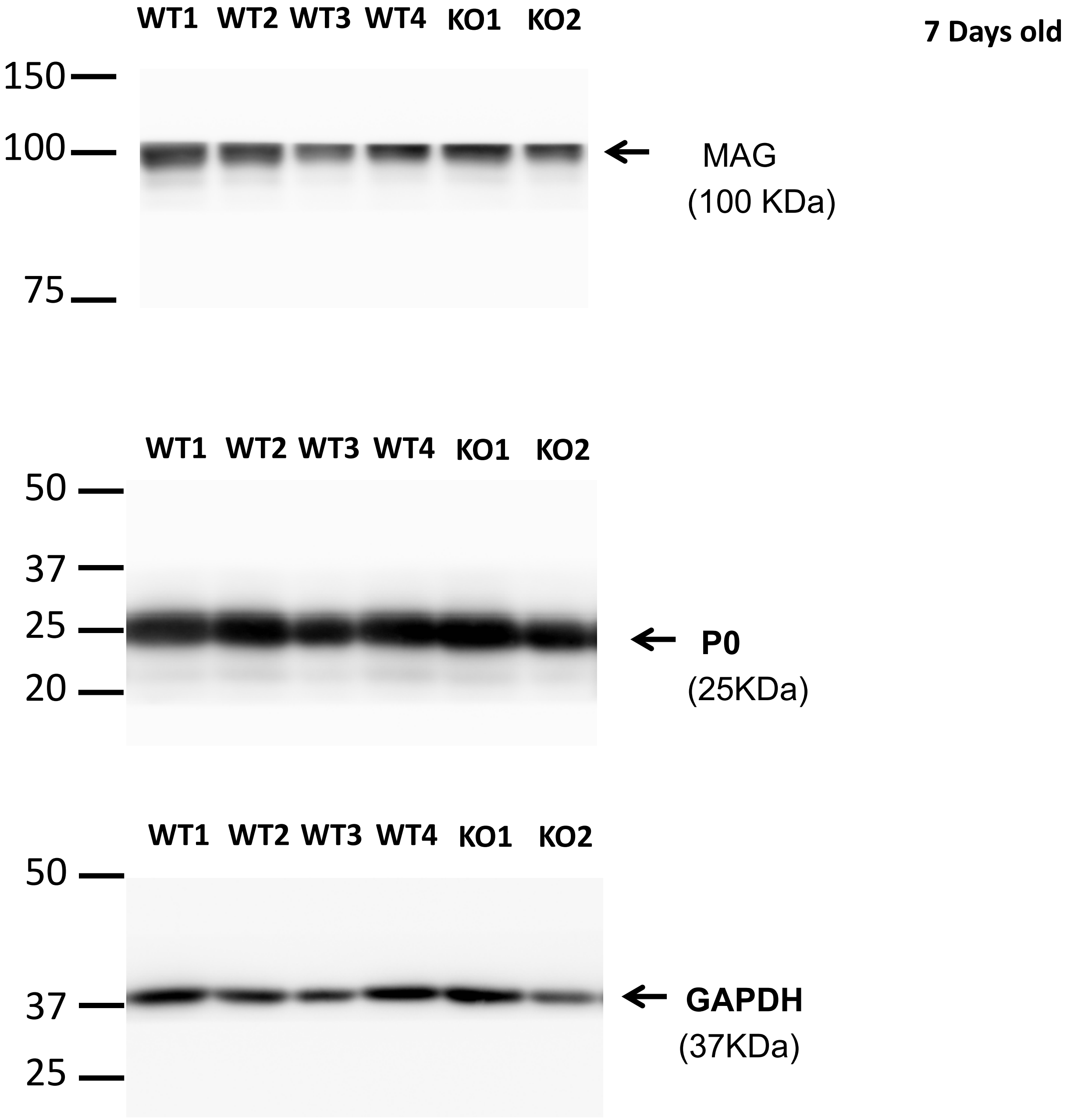

## Supplementary Fig 2

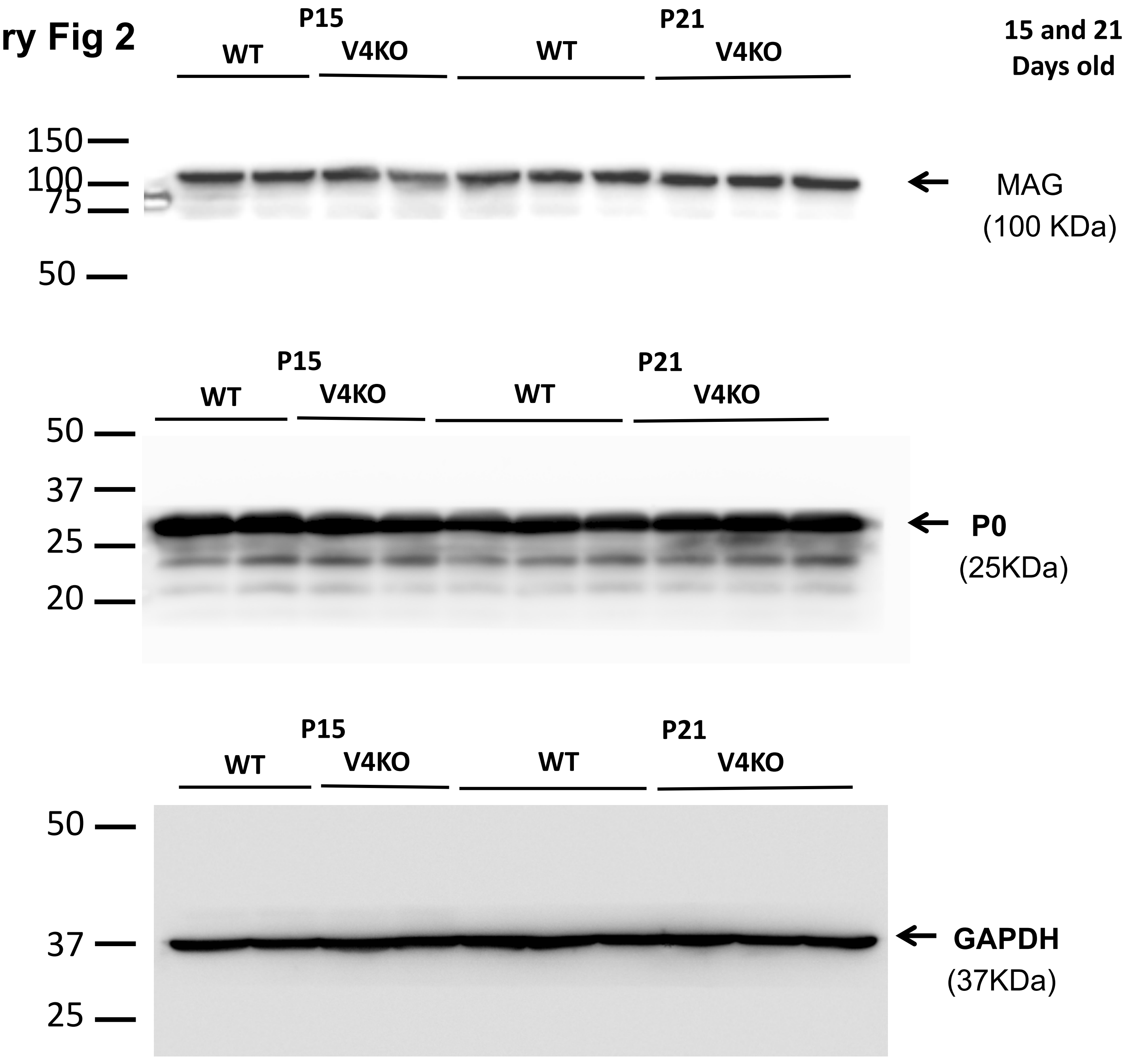

**Supplementary Fig 2**

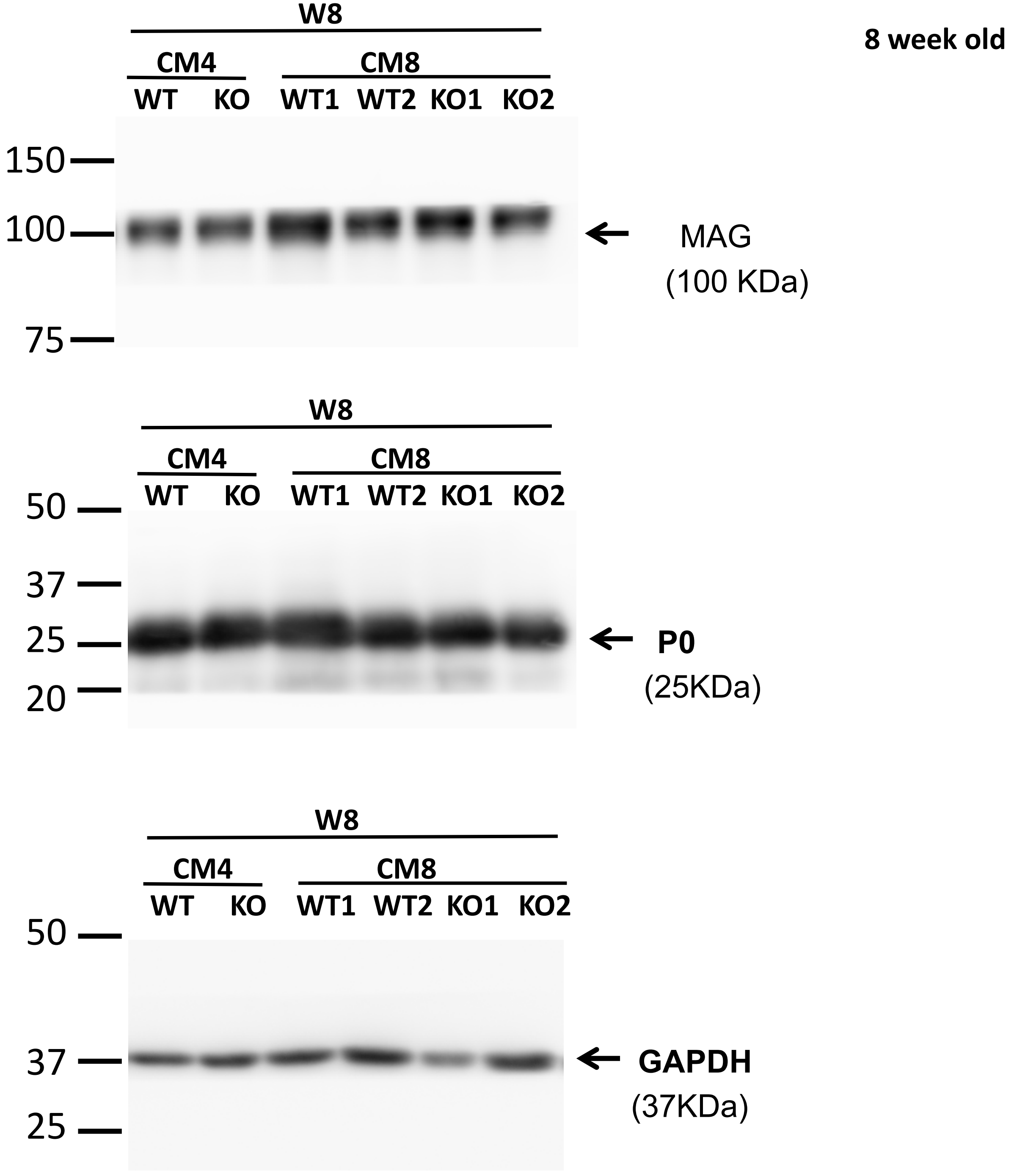

Supplement: Supplementary file 3 — Supplementary Data 1 [file 42003_2020_1444_MOESM3_ESM.pdf]
